# Supplementary material for: Interleukin-17A Causes Osteoarthritis-Like Transcriptional Changes in Human Osteoarthritis-Derived Chondrocytes and Synovial Fibroblasts In Vitro
Source: Front Immunol. 2021 May 12;12:676173. doi: 10.3389/fimmu.2021.676173 (PMC8153485; doi:10.3389/fimmu.2021.676173)
Supplement: Supplementary file 1 [file DataSheet_1.pdf]

Supplementary Material

Interleukin-17A Causes Osteoarthritis-Like Transcriptional Changes in Human Osteoarthritis-Derived Chondrocytes and Synovial Fibroblasts *In Vitro*

Jolet Y. Mimpen, Mathew J. Baldwin, Adam P. Cribbs, Martin Philpott, Andrew J. Carr, Stephanie G. Dakin, and Sarah J.B. Snelling

1 Supplementary Methods

1.1 Cell culture for p38 and NF-κB inhibitor treatment (regarding Supplementary Figures 8 and 9)

Chondrocytes and synovial fibroblasts were cultured and plated out in 96-well plates as described earlier. On the day of the experiment, cells were pre-treated for 2 hours with JSH-23, SB204580, or vehicle control (Table 4-4) before the addition of 10 ng/mL concentration of IL-17A for 24 hours. SB203580 inhibits p38 signalling by suppressing the activation of MAPKAP kinase-2 and inhibiting the phosphorylation of heat shock protein 27. JSH-23 inhibits NF-κB signalling by interfering with the nuclear localisation signals of p65. After 24 hours, cells were washed with PBS before being harvested in cells-to-cDNA Cell Lysis Buffer (Ambion Inc, Foster City, CA, USA), and transferred to a PCR plate for cells-to-cDNA synthesis. cDNA was prepared using a cells-to-cDNA kit following the manufacturer's instructions (Ambion).

2 Supplementary Figures

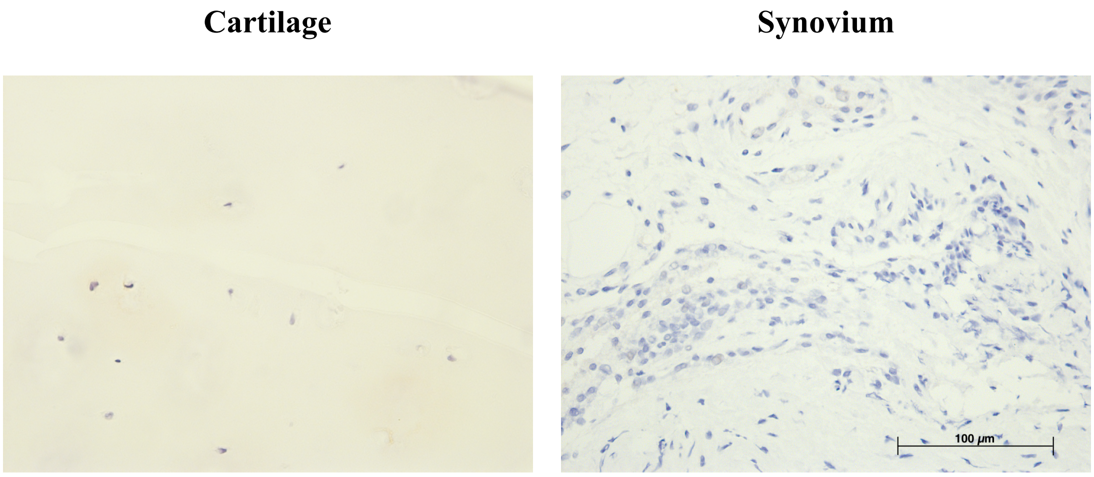

Supplementary Figure 1. Immunohistochemistry DAB staining with universal mouse negative control on cartilage or synovium. Antibodies were visualised with DAB (brown) and counterstained with haematoxylin (blue). Images were taken at 40x magnification

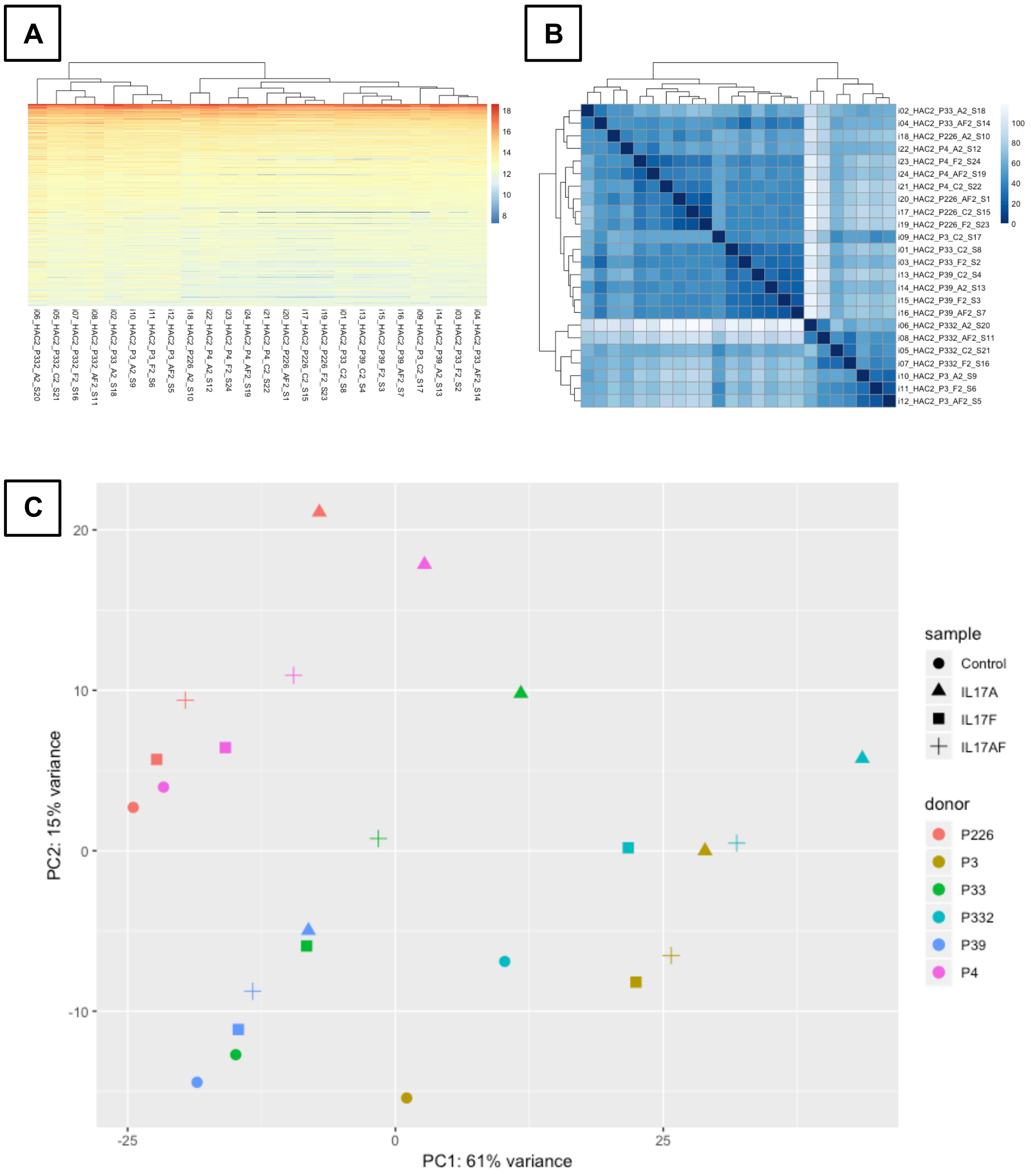

Supplementary Figure 2. Overview of different plots showing clustering of the chondrocytes. (A) Heatmap of the counts matrix for chondrocytes samples, blue shows low counts while red shows high counts. (B) Heatmap of the sample-to-sample distances, dark blue represents no to little difference while white represents large differences between samples. (C) Principle component analysis (PCA) plot for chondrocytes samples. Different sample types are represented with different shapes (circle = control, triangle = IL-17A, square = IL-17F, plus sign = IL-17AF) and different donors are represented with different colours (P226 = red, P3 = mustard, P33 = green, P332 = turquoise, P39 = blue, P4 = pink)

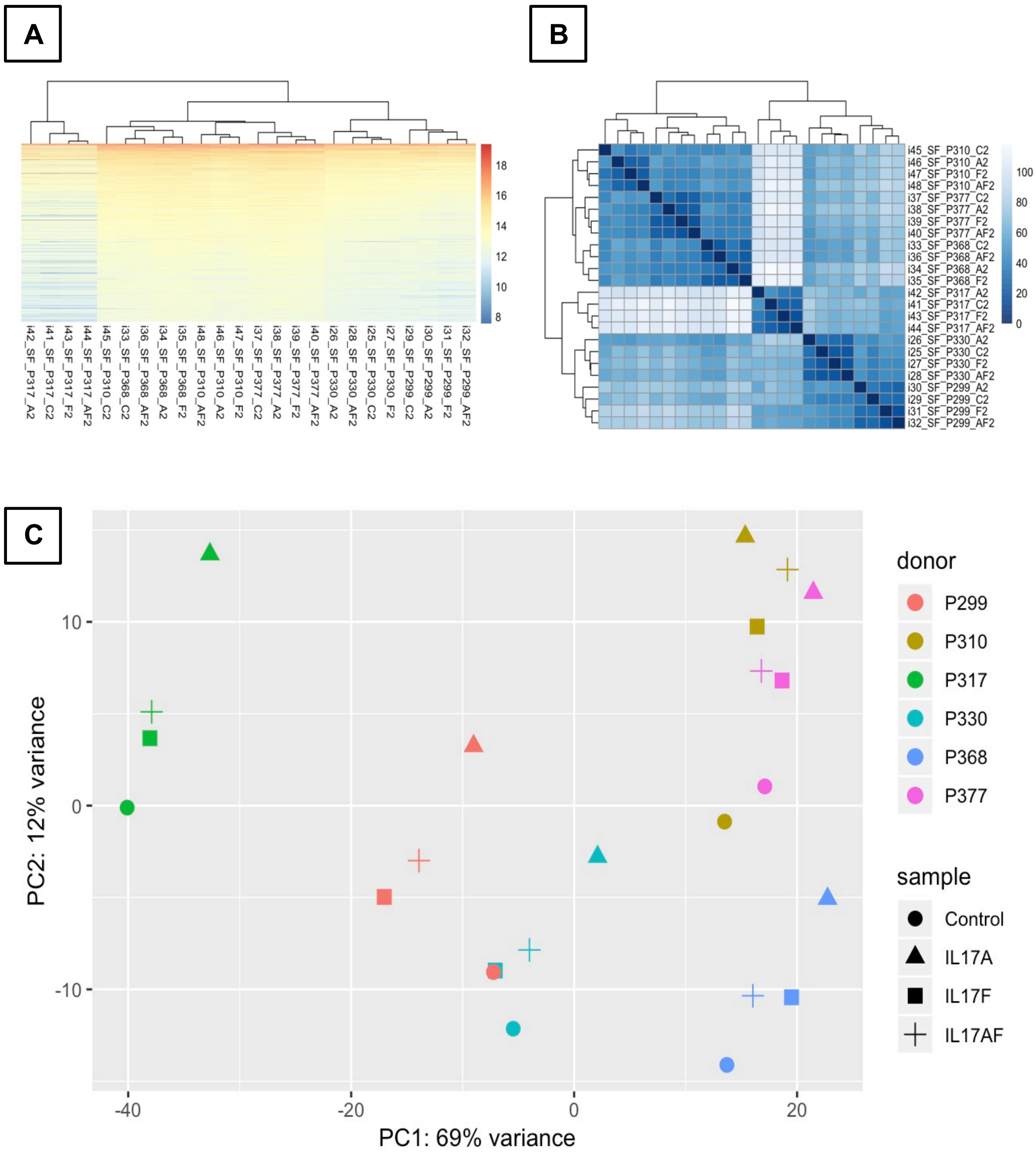

Supplementary Figure 3. Overview of different plots showing clustering of the synovial fibroblasts. (A) Heatmap of the counts matrix, blue shows low counts while red shows high counts. (B) Heatmap of the sample-to-sample distances, dark blue represents no to little difference while white represents large differences between samples. (C) Principle component analysis (PCA) plot for synovial fibroblast. Different sample types are represented with different shapes (circle = control, triangle = IL-17A, square = IL-17F, plus sign = IL-17AF) and different donors are represented with different colours (P299 = red, P310 = mustard, P317 = green, P330 = turquoise, P368 = blue, P377 = pink)

Supplementary Figure 4. Representative blots for the activation of p65 NF-κB, p38 MAP kinase, and ERK1/2 (p44/p42 MAP kinases) in chondrocytes and synovial fibroblasts by IL-17A, IL-17AF, and IL-17F (all 10 ng/ml) after 10 minutes, 30 minutes, 60 minutes, and 480 minutes (8 hours) of stimulation as can be seen in Figure 5 and Supplementary Figure 7. Blots were stained using vinculin (reference protein), and phosphorylated p38 or phosphorylated p65 NF-κB or phosphorylated ERK1/2, and total p38 or total p65 NF-κB or total ERK1/2, and blots were visualised with an enhanced chemiluminescent reagent.

(A) Protein lysate from end-stage chondrocytes stained for vinculin, phosphorylated p38, and total p38.

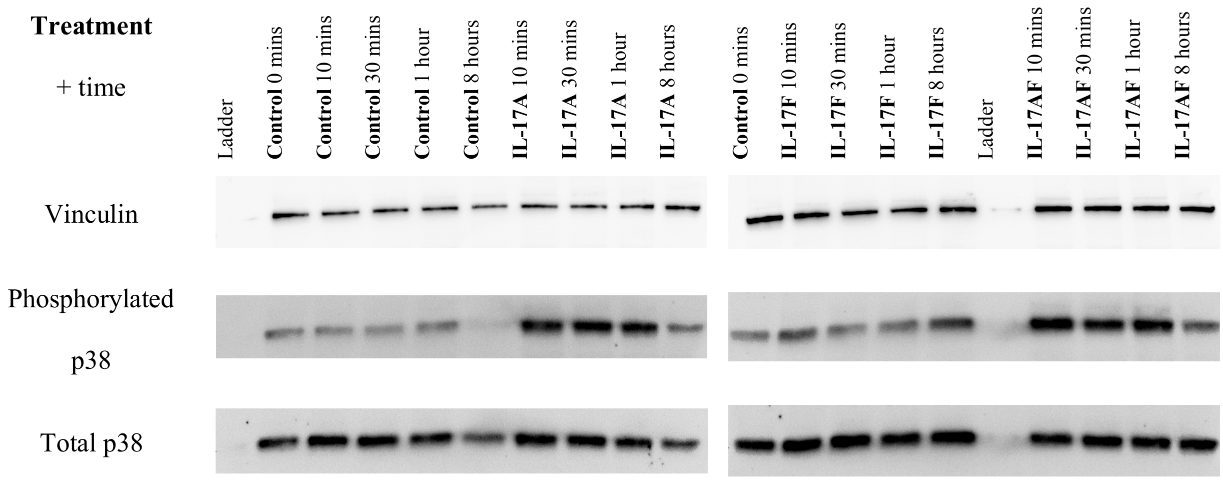

(B) Protein lysate from end-stage chondrocytes stained for vinculin, phosphorylated p65 NF-κB*, and total* p65 NF-κB.

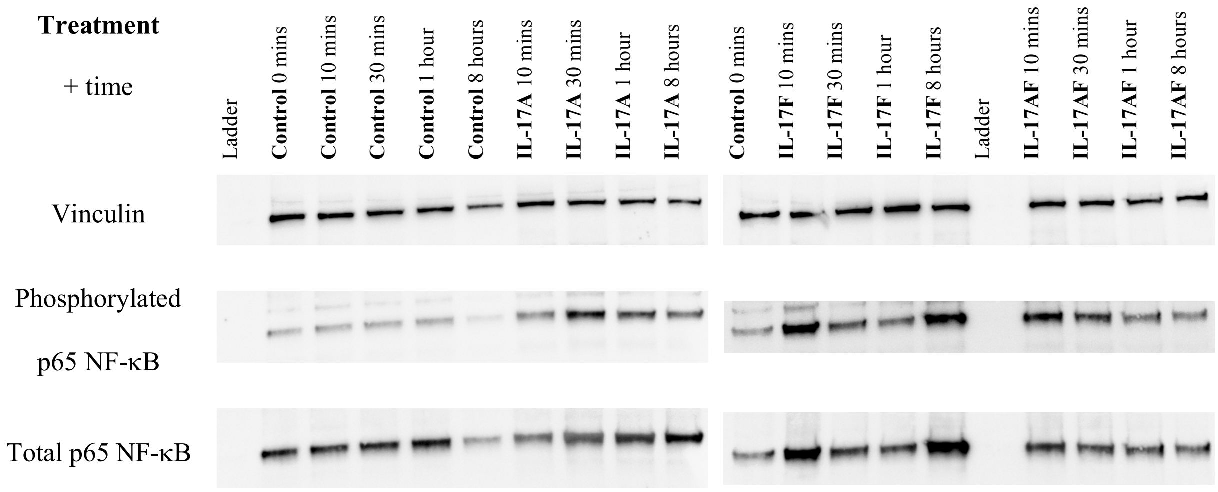

(C) Protein lysate from end-stage chondrocytes stained for vinculin, phosphorylated ERK1/2*, and total* ERK1/2.

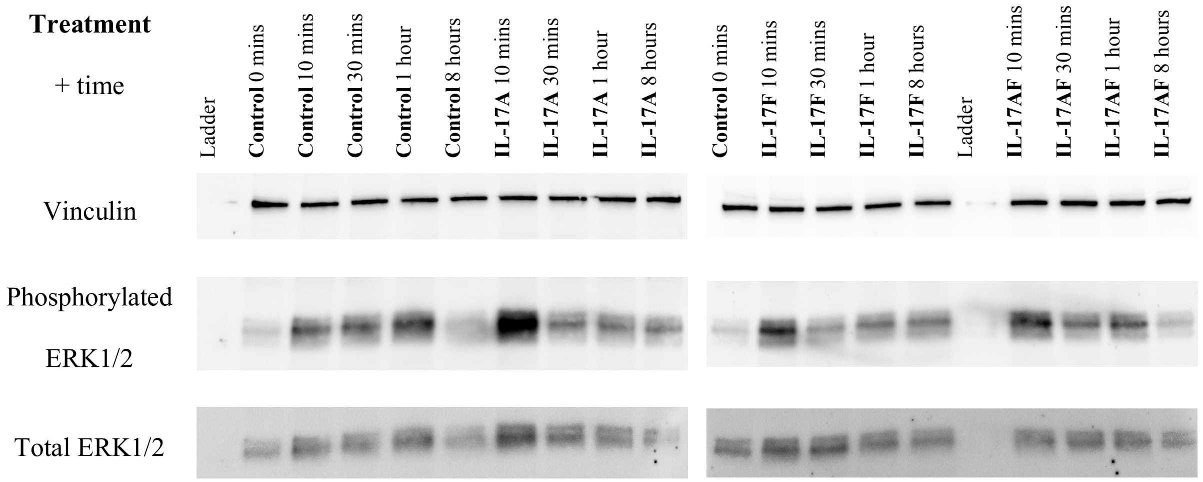

(D) Protein lysate from end-stage synovial fibroblasts stained for vinculin, phosphorylated p38, and total p38.

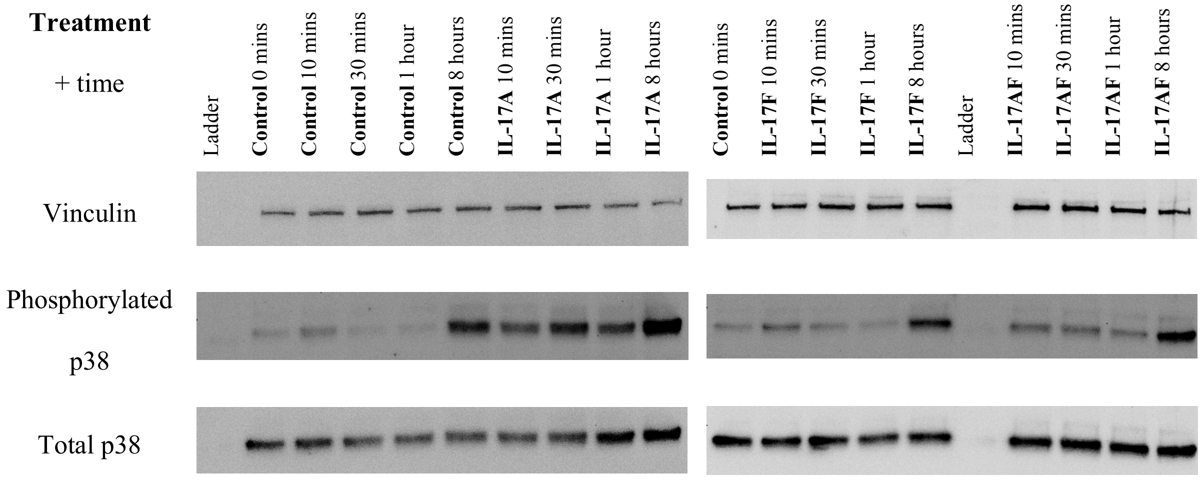

(E) Protein lysate from end-stage synovial fibroblasts treated with control or IL-17A stained for vinculin, phosphorylated p65 NF-κB*, and total* p65 NF-κB.

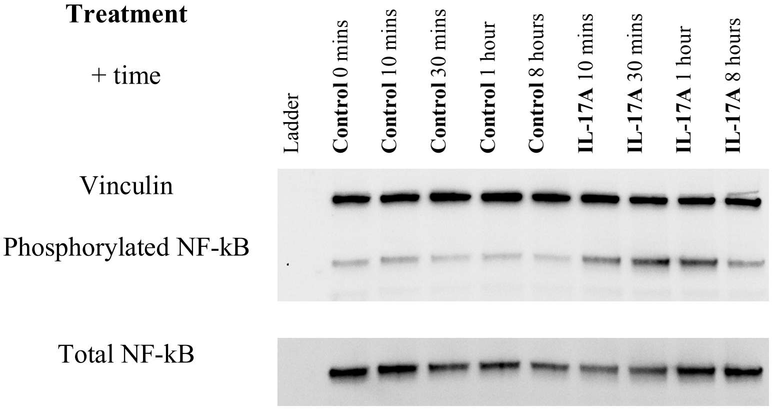

(F) Protein lysate from end-stage synovial fibroblasts treated with IL-17F and IL-17AF stained for vinculin, phosphorylated p65 NF-κB*, and total* p65 NF-κB.

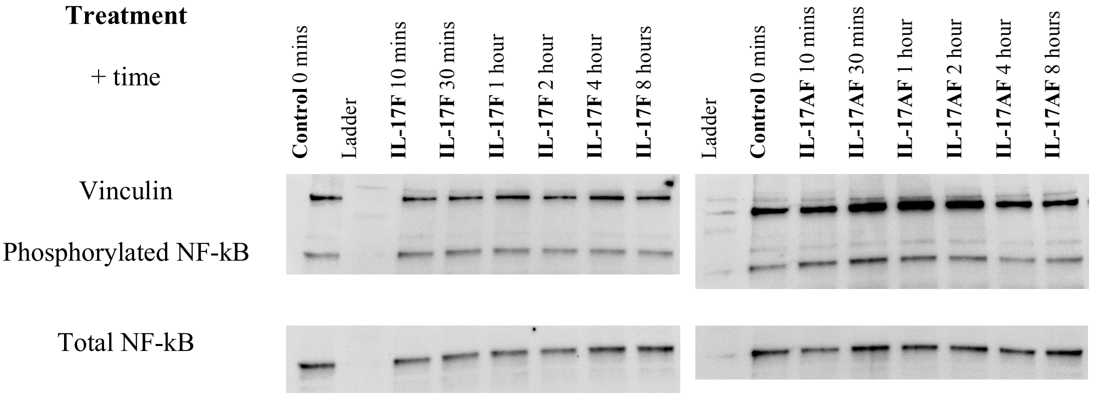

(G) Protein lysate from end-stage synovial fibroblasts stained for vinculin, phosphorylated ERK1/2*, and total* ERK1/2*.*

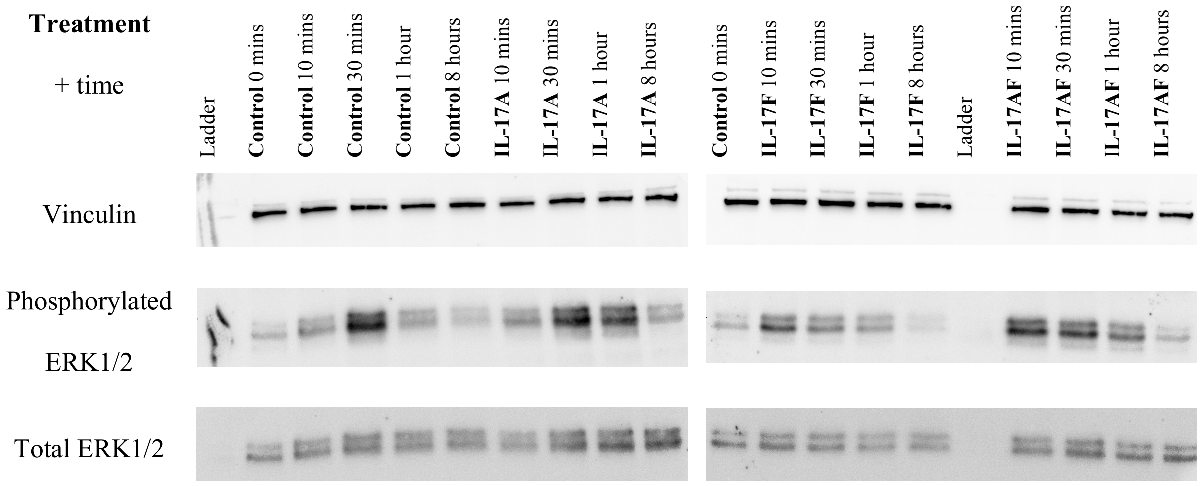

Chondrocytes

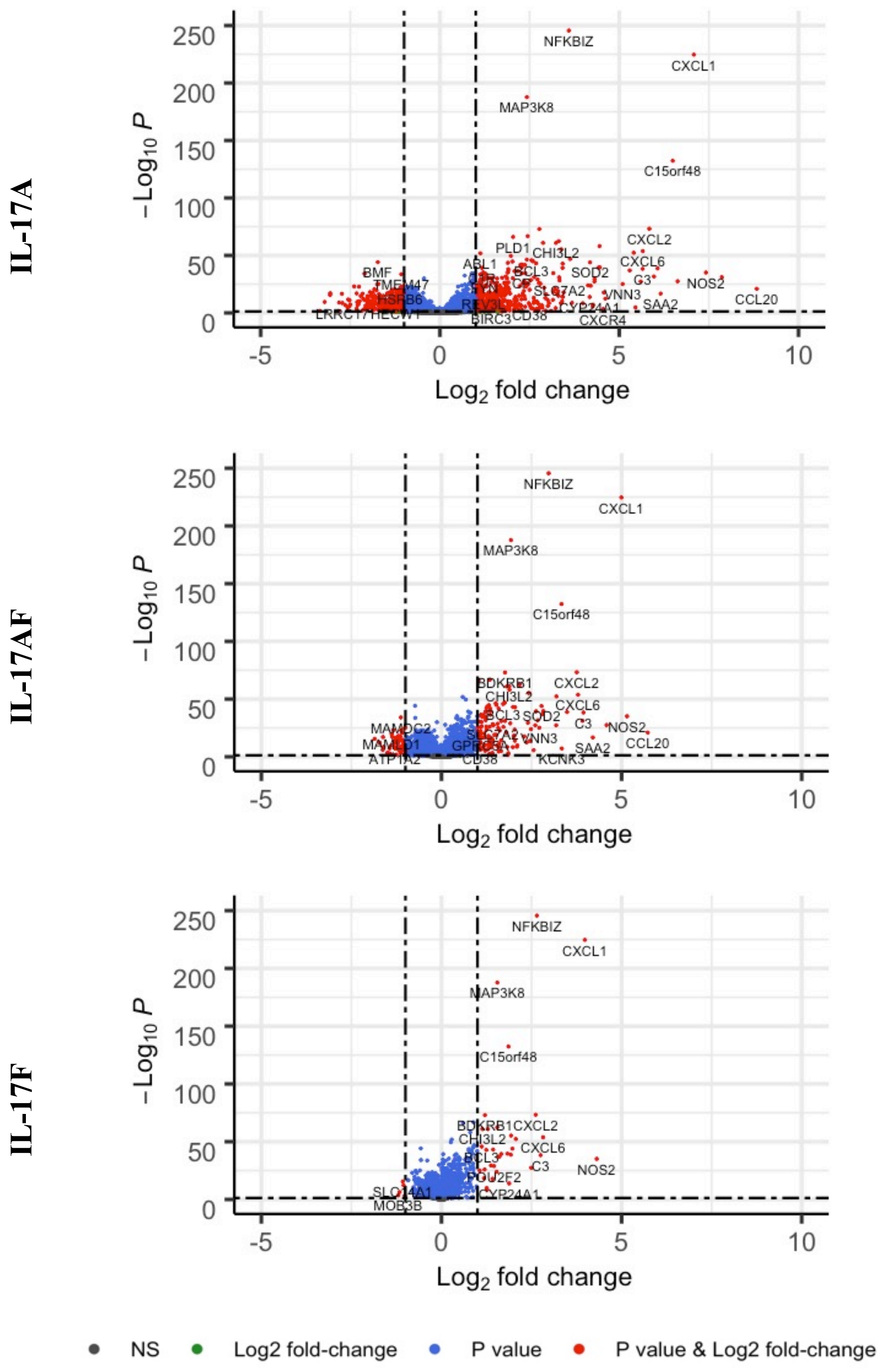

Supplementary Figure 5. Volcano plots showing the changes in gene expression in chondrocytes after treatment with IL-17A, IL-17AF, or IL-17F. **Grey** = not significant (NS); **green** = Log2 fold change of at least 1 or -1, but p>0.05; **blue** = p-value < 0.05, but log2 fold change >-1 and <1; **red** = p-value<0.05 and log2 fold change of at least -1 or +1. n=6

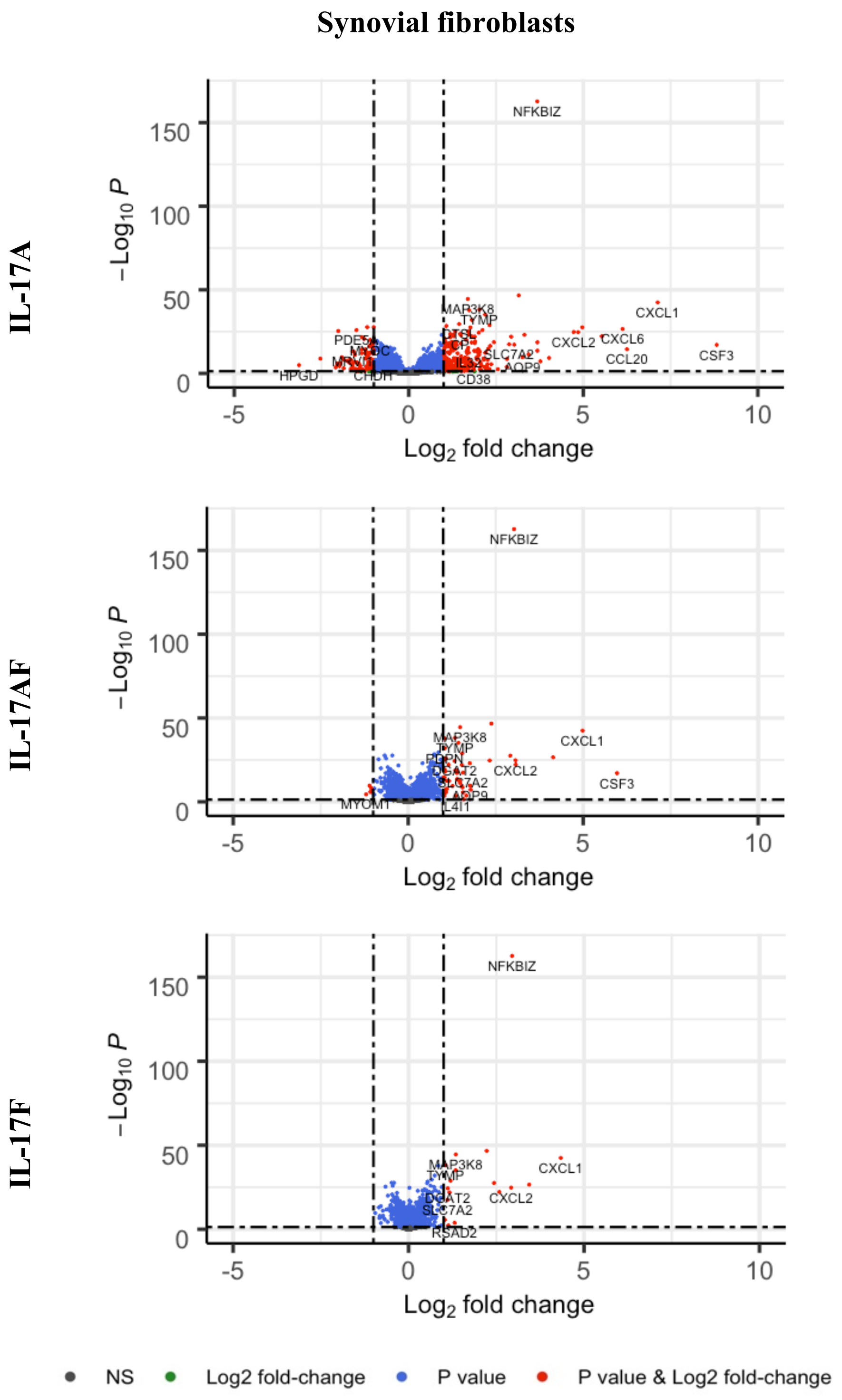

Supplementary Figure 6. Volcano plots showing the changes in gene expression in synovial fibroblasts after treatment with IL-17A, IL-17AF, or IL-17F. **Grey** = not significant (NS); **green** = Log2 fold change of at least 1 or -1, but p>0.05; **blue** = p-value < 0.05, but log2 fold change >-1 and <1; **red** = p-value<0.05 and log2 fold change of at least -1 or +1. n=6

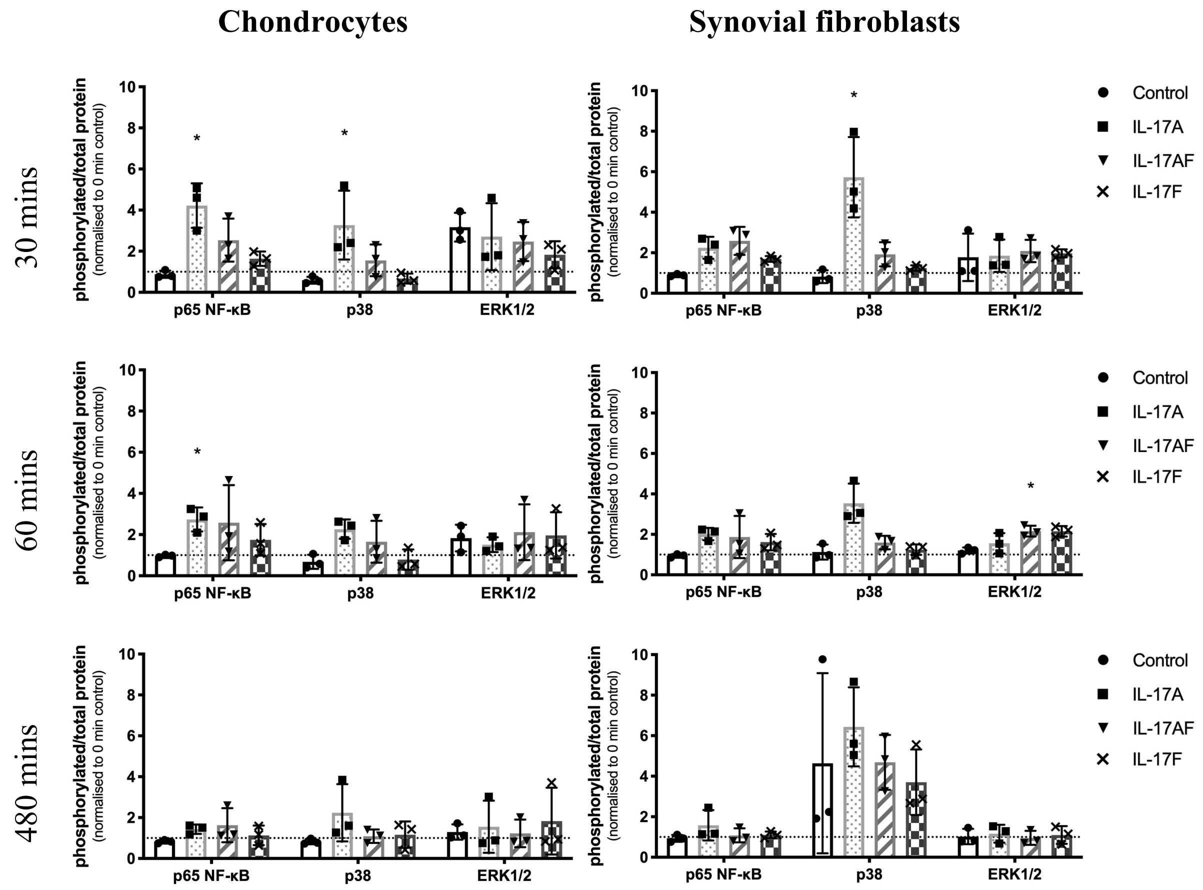

Supplementary Figure 7. Activation of p65 NF-κB, p38 MAP kinase, and ERK1/2 (p44/p42 MAP kinases) in chondrocytes and synovial fibroblasts by IL-17A, IL-17AF, and IL-17F (all 10 ng/ml) after 30 minutes, 60 minutes, and 480 minutes (8 hours) of stimulation. Relative ratio of phosphorylated protein over total protein compared to baseline control (0 mins). * = p<0.05, ** = p<0.01, *** = p<0.001, **** = p<0.0001. Friedman test for each intracellular protein with Dunn's multiple comparisons test. Mean±SD. N=3

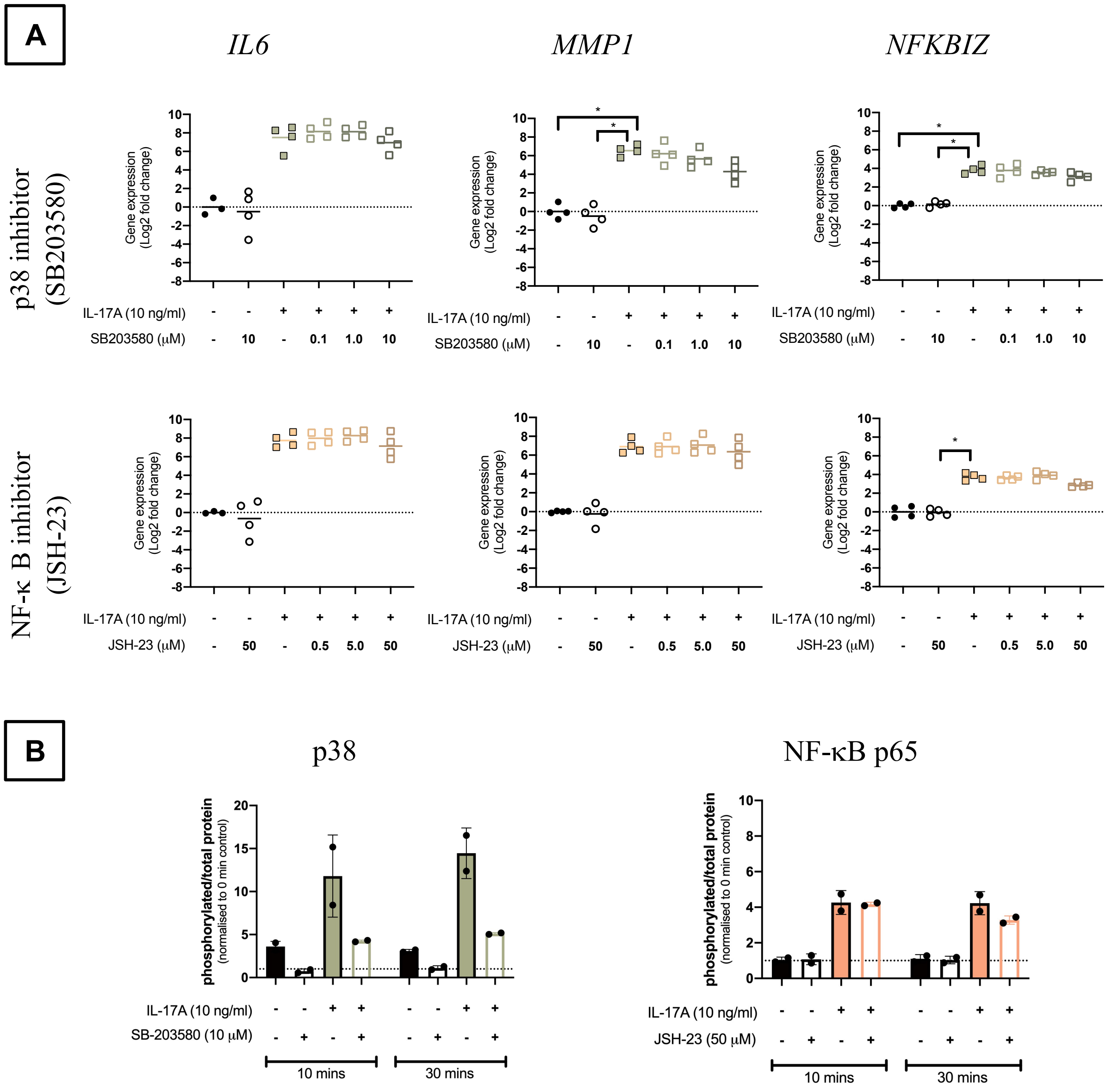

Supplementary Figure 8. Chondrocytes were pre-treated for 2 hours with SB203580 (**green**) (Sigma) at 0.1, 1.0, and 10 µM, JSH-23 (**orange**) (Sigma) at 0.5, 5.0, and 50 µM, or vehicle control before the addition of vehicle control or 10 ng/mL IL-17A for 24 hours. (A) mRNA expression of *IL6*, *MMP1*, and *NFKBIZ*. Gene expression was calculated using the ddCT-method using both *GAPDH* and *ACTB* as housekeepers. Gene expression is expressed as log2 fold change compared to control. Changes in gene expression were calculated comparing each treatment to IL-17A treatment alone. Kruskal-Wallis test with Dunn's multiple comparisons test (*IL6*). Friedman test with Dunn's multiple comparisons test (*CXCL1* and *MMP1*). Individual values and mean. N=4. (B) Activation of p38 or NF-κB p65 after 10 and 30 minutes after the start of IL-17A treatment. Relative ratio of phosphorylated protein over total protein compared to baseline control (0 mins). Mean±SD. N=2. * = p<0.05, ** = p<0.01, *** = p<0.001

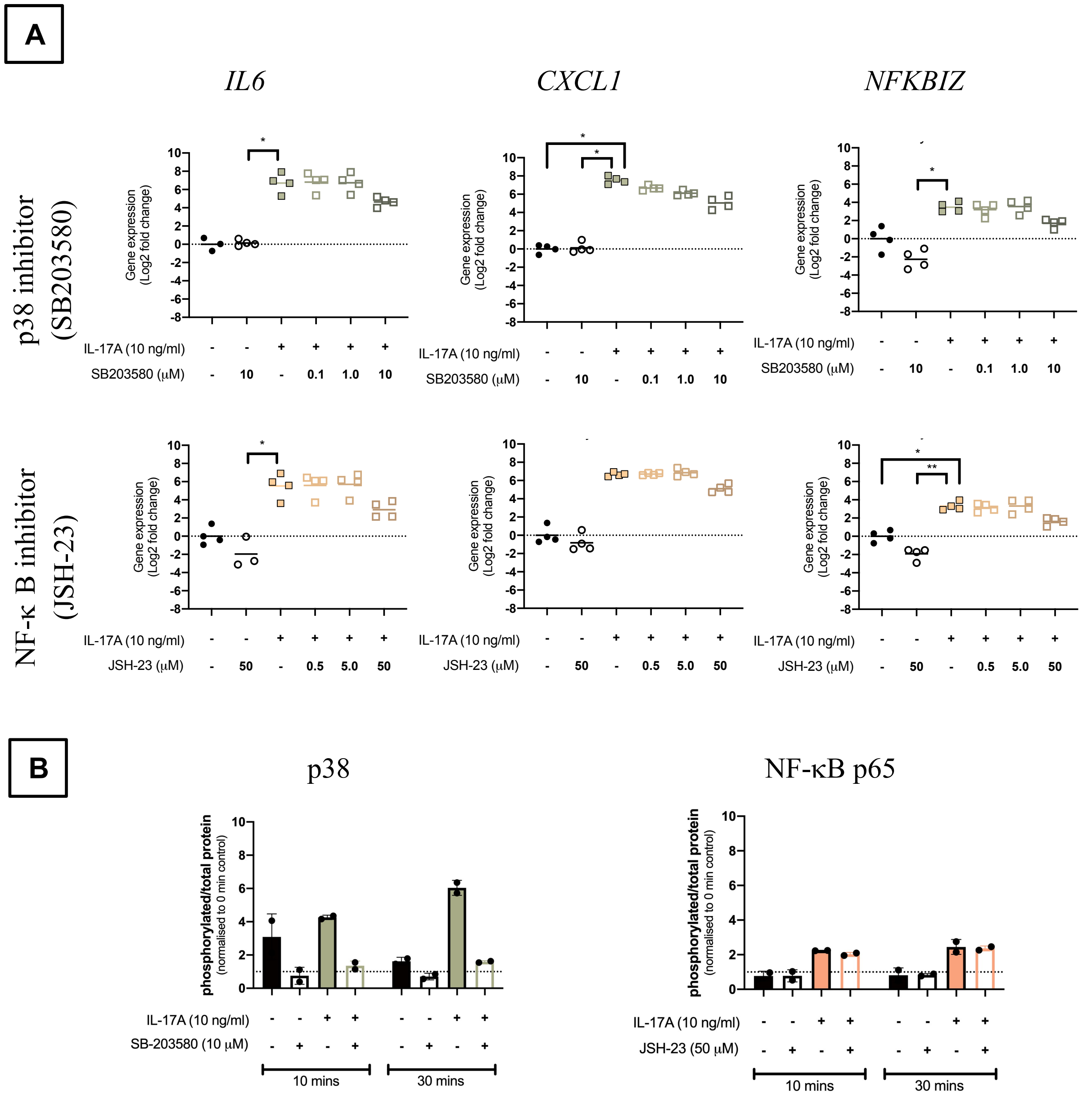

Supplementary Figure 9. Synovial fibroblasts were pre-treated for 2 hours with SB203580 (**green**) (Sigma) at 0.1, 1.0, and 10 µM, JSH-23 (**orange**) (Sigma) at 0.5, 5.0, and 50 µM, or vehicle control before the addition of vehicle control or 10 ng/mL IL-17A for 24 hours. (A) mRNA expression of *IL6*, *CXCL1*, and *NFKBIZ*. Gene expression was calculated using the ddCT-method using both *GAPDH* and *ACTB* as housekeepers. Gene expression is expressed as log2 fold change compared to control. Changes in gene expression were calculated comparing each treatment to IL-17A treatment alone. Kruskal-Wallis test with Dunn's multiple comparisons test (*IL6*). Friedman test with Dunn's multiple comparisons test (*CXCL1* and *NFKBIZ*). Individual values and mean. N=4. (B) Activation of p38 or NF-κB p65 after 10 and 30 minutes after the start of IL-17A treatment. Relative ratio of phosphorylated protein over total protein compared to baseline control (0 mins). Mean±SD. N=2. * = p<0.05, ** = p<0.01, *** = p<0.001.

3 Supplementary Tables

Supplementary Table 1. Primary antibodies used for histology, including the target, isotype, supplier, reference number, and dilution at which each antibody was used.

Target	Isotype	Supplier	Ref number	Dilution
Human IL-17RA	Monoclonal mouse IgG2B	R&D Systems, Abingdon, UK	MAB1771	1:300
Human IL-17RC	Monoclonal mouse IgG2B	R&D Systems	MAB22691	1:2000
Universal negative control mouse	Cocktail of mouse IgG1, IgG2a, IgG2b, IgG3, and IgM	Dako, Glostrup, Denmark	IR750	Ready to use

Supplementary Table 2. Primary and secondary antibodies used for western blot experiments, including target, supplier, reference number, and the dilution at which each antibody was used.

Target	Supplier	Ref number	Dilution
Vinculin (reference protein)	Cell Signalling Technology, Danvers, MA, USA	13901	1:1000
phospho-ERK1/2	Cell Signalling Technology	4370	1:2000
ERK1/2	Cell Signalling Technology	4695	1:1000
phospho-p65 NF-κB	Cell Signalling Technology	3033	1:1000
p65 NF-κB	Cell Signalling Technology	8242	1:1000
phospho-p38	Cell Signalling Technology	4511	1:1000
p38	Cell Signalling Technology	8690	1:1000
Anti-rabbit IgG, HRP-linked	Cell Signalling Technology	7074	1:1000 (phospho-antibodies) 1:2000 (total-antibodies)

Supplementary Table 3. Overview of primers, including the source of the primers, and the forward and reverse primer sequences where available.

Target	Supplier	Forward	Reverse
ACTB	Designed in-house	CCTGGCACCCAGCACAAT	GCCGATCCACACGGAGTACT
CXCL1	Designed in-house	GGCGGAAAGCTTGCCTCAATCCTG	GCCTCCTTCAGGAACAGCCACCA
GAPDH	Designed in-house	GAAGGTTGAAGGTCGGAGTC	GAAGATGGTGATGGGATTTC
IL17RA	Primerdesign Ltd	TGAGCACATGCACCACATAC	TTAAGGTTGCGTAGAGTGAGTG
IL17RC	Primerdesign Ltd	AGTCGTGCTCTCCTTCCAG	AGATTCGTACCTTCACTCCCTAG
IL6	Primerdesign Ltd	GCAGAAAACAACCTGAACCTT	ACCTCAAACTCCAAAAGACCA
MMP1	Primerdesign Ltd	GCACTGAGAAAGAAGACAAAGG	CTAAGTCCACATCTTGCTCTTG
NFKBIZ	Qiagen	*Proprietary sequence*	*Proprietary sequence*
PDPN	Primerdesign Ltd	CCGAAGATGATGTGGTGACTC	CGATGCGAATGCCTGTTACA

Supplementary Table 4. Overview of differentially expressed genes in chondrocytes between vehicle control and IL-17 treatment (10 ng/ml) with an adjusted p-value of <0.05. Green boxes note logarithmic fold changes (LFC) of at least +1 and red boxes note LFC of at least -1, while blank boxes show an LFC -1 and 1. Genes are sorted alphabetically on SYMBOL gene name.

Ensemble gene name	SYMBOL gene name	Adjusted p-value	Log2 Fold Change (LFC)		
			IL-17A	IL-17F	IL-17AF
ENSG00000183044	*ABAT*	3.74E-11	-1.073	-0.357	-0.744
ENSG00000165029	*ABCA1*	2.28E-17	1.199	0.187	0.450
ENSG00000167972	*ABCA3*	4.89E-10	-1.475	-0.491	-0.923
ENSG00000005471	*ABCB4*	7.13E-09	-1.011	-0.364	-0.989
ENSG00000069431	*ABCC9*	7.20E-05	1.850	0.005	0.053
ENSG00000143994	*ABHD1*	5.23E-03	-1.571	-0.026	-0.177
ENSG00000011198	*ABHD5*	3.70E-11	1.051	0.039	0.405
ENSG00000097007	*ABL1*	1.35E-52	1.128	0.285	0.589
ENSG00000240303	*ACAD11*	3.42E-18	-1.009	-0.309	-0.566
ENSG00000115361	*ACADL*	1.18E-03	-1.519	-0.026	-0.234
ENSG00000144476	*ACKR3*	5.73E-31	2.033	0.645	1.110
ENSG00000129048	*ACKR4*	9.29E-10	-2.812	-0.022	-1.313
ENSG00000122729	*ACO1*	7.38E-16	1.051	0.201	0.425
ENSG00000068366	*ACSL4*	7.02E-18	1.712	0.049	0.521
ENSG00000107796	*ACTA2*	4.53E-08	-1.184	-0.328	-0.727
ENSG00000159251	*ACTC1*	1.80E-17	-2.156	-0.923	-1.205
ENSG00000148848	*ADAM12*	5.00E-18	1.387	0.269	0.642
ENSG00000042980	*ADAM28*	1.27E-14	3.424	0.026	2.038
ENSG00000138316	*ADAMTS14*	2.34E-17	-2.734	-0.926	-1.281
ENSG00000166106	*ADAMTS15*	8.81E-09	-1.549	-0.684	-1.166
ENSG00000049192	*ADAMTS6*	4.16E-04	1.158	0.015	0.109
ENSG00000163638	*ADAMTS9*	2.97E-05	1.616	0.013	0.347
ENSG00000173567	*ADGRF3*	1.50E-02	-1.032	-0.027	-0.256
ENSG00000198099	*ADH4*	1.85E-02	1.357	0.018	0.207
ENSG00000196344	*ADH7*	8.39E-04	-1.117	-0.019	-0.178
ENSG00000128271	*ADORA2A*	5.07E-03	1.074	0.001	0.096
ENSG00000150594	*ADRA2A*	1.96E-09	-2.284	-0.040	-1.134
ENSG00000111863	*ADTRP*	1.73E-06	1.270	0.052	0.545
ENSG00000144218	*AFF3*	8.87E-08	-2.297	-0.025	-0.980
ENSG00000187546	*AGMO*	1.97E-02	-1.307	-0.022	-0.183
ENSG00000026652	*AGPAT4*	1.57E-10	1.227	0.047	0.421
ENSG00000162433	*AK4*	5.46E-09	1.092	0.102	0.484

ENSG00000154027	*AK5*	2.21E-12	-2.558	-0.040	-1.029
ENSG00000131016	*AKAP12*	1.66E-14	-1.242	-0.704	-1.268
ENSG00000151320	*AKAP6*	4.58E-09	-1.153	-0.478	-1.004
ENSG00000227471	*AKR1B15*	2.50E-03	1.352	0.027	0.852
ENSG00000187134	*AKR1C1*	3.14E-07	1.965	0.750	1.276
ENSG00000151632	*AKR1C2*	2.03E-07	1.269	0.644	1.006
ENSG00000144908	*ALDH1L1*	4.75E-02	-1.196	-0.019	-0.063
ENSG00000112294	*ALDH5A1*	6.15E-08	-1.341	-0.070	-0.821
ENSG00000133805	*AMPD3*	4.23E-12	1.453	0.045	0.424
ENSG00000091879	*ANGPT2*	3.13E-10	-1.587	-0.040	-0.828
ENSG00000136859	*ANGPTL2*	7.50E-14	1.008	0.308	0.677
ENSG00000167772	*ANGPTL4*	3.14E-09	1.289	0.360	0.858
ENSG00000165887	*ANKRD2*	1.50E-02	-1.636	-0.019	-0.122
ENSG00000089847	*ANKRD24*	1.18E-03	-1.049	-0.035	-0.838
ENSG00000138772	*ANXA3*	5.96E-03	-1.096	0.001	-0.096
ENSG00000138356	*AOX1*	2.39E-09	1.343	0.033	0.397
ENSG00000154856	*APCDD1*	3.94E-09	1.367	0.395	0.864
ENSG00000171388	*APLN*	3.82E-06	2.090	0.003	0.113
ENSG00000084674	*APOB*	2.51E-02	-1.071	-0.015	-0.210
ENSG00000128284	*APOL3*	4.77E-13	1.100	0.283	0.565
ENSG00000136044	*APPL2*	3.06E-23	-1.084	-0.345	-0.595
ENSG00000165272	*AQP3*	1.33E-14	-1.623	-0.083	-0.400
ENSG00000165269	*AQP7*	2.07E-10	-2.289	-0.602	-1.063
ENSG00000103569	*AQP9*	1.60E-02	3.101	0.005	0.045
ENSG00000169083	*AR*	1.38E-12	-1.150	-0.415	-0.719
ENSG00000146376	*ARHGAP18*	2.83E-05	-1.089	-0.038	-0.614
ENSG00000137727	*ARHGAP20*	1.77E-08	-1.442	-0.053	-0.853
ENSG00000111348	*ARHGDIB*	7.34E-06	-1.345	-0.482	-0.334
ENSG00000183111	*ARHGEF37*	1.34E-07	-1.136	-0.283	-0.733
ENSG00000116017	*ARID3A*	1.49E-19	1.072	0.129	0.485
ENSG00000169126	*ARMC4*	2.72E-03	-1.309	-0.027	-0.391
ENSG00000113369	*ARRDC3*	4.44E-16	1.691	0.060	0.606
ENSG00000099889	*ARVCF*	4.56E-11	-1.192	-0.077	-0.524
ENSG00000110881	*ASIC1*	1.21E-06	-1.006	-0.072	-0.563
ENSG00000108381	*ASPA*	7.01E-03	-1.037	-0.027	-0.359
ENSG00000106819	*ASPN*	1.12E-15	-1.910	-0.606	-1.141
ENSG00000130707	*ASS1*	1.20E-23	1.190	0.346	0.735
ENSG00000148219	*ASTN2*	8.34E-04	-1.119	-0.028	-0.440
ENSG00000162772	*ATF3*	1.50E-08	1.265	0.101	1.044
ENSG00000018625	*ATP1A2*	2.36E-08	-2.275	-0.755	-1.289

ENSG00000070961	*ATP2B1*	7.01E-08	1.003	0.076	0.355
ENSG00000132932	*ATP8A2*	1.27E-02	-0.792	-0.019	-1.255
ENSG00000104043	*ATP8B4*	9.96E-05	1.323	0.023	0.374
ENSG00000107518	*ATRNL1*	9.38E-08	-1.002	-0.285	-0.624
ENSG00000119986	*AVPI1*	9.65E-19	1.141	0.359	0.722
ENSG00000162630	*B3GALT2*	2.98E-10	-3.217	-0.011	-0.179
ENSG00000086062	*B4GALT1*	1.19E-15	1.001	0.105	0.436
ENSG00000164929	*BAALC*	4.05E-04	-1.115	-0.011	-0.211
ENSG00000095739	*BAMBI*	6.21E-10	-1.034	-0.039	-0.270
ENSG00000176788	*BASP1*	1.07E-13	1.150	0.431	0.836
ENSG00000156127	*BATF*	4.55E-04	1.203	0.003	0.105
ENSG00000140379	*BCL2A1*	3.52E-03	2.286	0.001	0.042
ENSG00000069399	*BCL3*	1.74E-46	2.557	1.118	1.713
ENSG00000113916	*BCL6*	9.25E-15	1.181	0.252	0.600
ENSG00000100739	*BDKRB1*	9.95E-74	2.772	1.207	1.764
ENSG00000168398	*BDKRB2*	5.42E-23	1.883	0.723	1.086
ENSG00000176697	*BDNF*	1.35E-04	1.121	0.016	0.198
ENSG00000167995	*BEST1*	7.21E-31	1.871	0.425	0.971
ENSG00000134107	*BHLHE40*	2.26E-08	1.342	0.067	0.742
ENSG00000123095	*BHLHE41*	7.70E-24	1.313	0.123	0.421
ENSG00000015475	*BID*	3.25E-24	1.017	0.288	0.452
ENSG00000023445	*BIRC3*	7.20E-05	1.436	0.007	0.072
ENSG00000104081	*BMF*	6.54E-45	-1.733	-0.574	-0.733
ENSG00000125845	*BMP2*	4.94E-03	1.038	0.010	0.185
ENSG00000153162	*BMP6*	6.62E-13	2.375	0.031	1.093
ENSG00000078725	*BRINP1*	6.40E-05	1.137	0.023	0.207
ENSG00000133639	*BTG1*	1.53E-15	1.059	0.386	0.651
ENSG00000154493	*C10orf90*	6.09E-14	1.446	0.791	1.073
ENSG00000120055	*C10orf95*	1.61E-02	-1.150	-0.016	-0.148
ENSG00000110665	*C11orf21*	1.46E-02	-1.137	-0.004	-0.130
ENSG00000174370	*C11orf45*	4.37E-07	-2.072	-1.161	-1.111
ENSG00000149300	*C11orf52*	3.30E-05	-1.092	-0.380	-0.853
ENSG00000187479	*C11orf96*	1.00E-21	3.216	0.948	1.801
ENSG00000235162	*C12orf75*	1.06E-23	-1.036	-0.370	-0.537
ENSG00000166920	*C15orf48*	4.24E-133	6.492	1.860	3.333
ENSG00000132016	*C19orf57*	1.87E-03	-1.231	-0.032	-0.123
ENSG00000106392	*C1GALT1*	4.18E-37	1.908	0.703	1.091
ENSG00000173918	*C1QTNF1*	6.70E-21	1.844	0.027	0.602
ENSG00000145861	*C1QTNF2*	6.45E-10	-1.790	-0.048	-0.804
ENSG00000163145	*C1QTNF7*	4.42E-10	-2.307	-0.748	-1.415

ENSG00000205863	*C1QTNF9B*	8.86E-03	-1.211	-0.020	-0.088
ENSG00000159403	*C1R*	6.20E-40	1.174	0.413	0.742
ENSG00000139178	*C1RL*	7.07E-35	1.241	0.455	0.750
ENSG00000198535	*C2CD4A*	1.97E-06	4.262	0.015	2.558
ENSG00000205502	*C2CD4B*	1.51E-05	5.453	0.009	0.089
ENSG00000125730	*C3*	4.39E-39	5.653	2.751	3.940
ENSG00000197405	*C5AR1*	5.40E-05	1.264	0.016	0.141
ENSG00000178776	*C5orf46*	4.35E-02	1.714	0.007	0.056
ENSG00000074410	*CA12*	3.53E-35	2.385	0.658	1.177
ENSG00000107159	*CA9*	3.95E-04	1.660	0.024	0.215
ENSG00000183346	*CABCOCO1*	3.51E-08	-1.205	-0.119	-0.604
ENSG00000070808	*CAMK2A*	1.08E-05	-1.522	-0.052	-1.159
ENSG00000058404	*CAMK2B*	4.19E-04	-2.109	-0.023	-0.775
ENSG00000105519	*CAPS*	9.33E-10	-1.012	-0.134	-0.419
ENSG00000204682	*CASC10*	4.69E-08	-1.645	-0.044	-1.292
ENSG00000168497	*CAVIN2*	5.99E-14	-2.012	-0.077	-1.198
ENSG00000150636	*CCDC102B*	1.60E-03	-1.307	-0.018	-0.121
ENSG00000168491	*CCDC110*	1.67E-07	-1.046	-0.402	-0.800
ENSG00000198003	*CCDC151*	2.12E-02	-1.032	-0.024	-0.216
ENSG00000166510	*CCDC68*	1.39E-02	1.033	0.021	0.115
ENSG00000055813	*CCDC85A*	3.05E-12	-1.225	-0.043	-0.536
ENSG00000181374	*CCL13*	4.54E-02	1.739	0.001	0.035
ENSG00000108691	*CCL2*	1.17E-24	4.129	1.530	2.484
ENSG00000115009	*CCL20*	1.03E-21	8.836	0.022	5.726
ENSG00000213927	*CCL27*	1.11E-02	-1.397	-0.011	-0.136
ENSG00000108688	*CCL7*	3.78E-13	4.614	0.022	2.363
ENSG00000108700	*CCL8*	4.55E-28	5.598	0.010	2.603
ENSG00000135083	*CCNJL*	1.29E-02	-1.102	-0.031	-0.175
ENSG00000163823	*CCR1*	1.52E-02	1.585	0.005	0.088
ENSG00000126353	*CCR7*	1.31E-15	2.201	0.785	0.912
ENSG00000158473	*CD1D*	4.79E-19	3.168	1.179	2.009
ENSG00000174807	*CD248*	3.03E-02	-1.244	-0.008	-0.109
ENSG00000004468	*CD38*	7.16E-08	2.508	0.009	1.078
ENSG00000085117	*CD82*	1.69E-15	1.081	0.156	0.464
ENSG00000074276	*CDHR2*	4.85E-02	-1.122	-0.020	-0.076
ENSG00000138395	*CDK15*	1.80E-02	-2.317	-0.011	-0.071
ENSG00000138769	*CDKL2*	2.99E-03	-1.391	-0.023	-0.617
ENSG00000129596	*CDO1*	3.01E-11	1.199	0.416	0.669
ENSG00000172216	*CEBPB*	2.40E-32	1.838	0.761	1.227
ENSG00000221869	*CEBPD*	7.11E-44	2.682	1.434	1.985

ENSG00000184524	*CEND1*	2.02E-03	1.365	0.017	0.690
ENSG00000188452	*CERKL*	1.47E-08	-1.523	-0.615	-0.657
ENSG00000105792	*CFAP69*	1.68E-18	1.933	0.051	0.917
ENSG00000243649	*CFB*	1.31E-22	2.906	0.030	0.834
ENSG00000128849	*CGNL1*	2.59E-07	1.058	0.031	0.204
ENSG00000138135	*CH25H*	3.44E-26	2.801	0.030	1.085
ENSG00000100399	*CHADL*	5.07E-07	-1.652	-0.026	-0.516
ENSG00000016391	*CHDH*	1.53E-15	-1.299	-0.283	-0.471
ENSG00000183765	*CHEK2*	1.11E-10	1.379	0.479	0.809
ENSG00000133048	*CHI3L1*	2.71E-44	1.892	0.758	1.273
ENSG00000064886	*CHI3L2*	1.22E-61	3.236	1.137	1.880
ENSG00000255112	*CHMP1B*	4.05E-18	1.514	0.451	0.876
ENSG00000128656	*CHN1*	1.12E-12	-1.419	-0.039	-0.429
ENSG00000054938	*CHRDL2*	6.11E-05	1.706	0.011	0.192
ENSG00000171310	*CHST11*	2.96E-15	1.547	0.042	0.508
ENSG00000163815	*CLEC3B*	2.87E-05	-1.061	-0.063	-0.451
ENSG00000169583	*CLIC3*	2.64E-07	-1.020	-0.330	-0.436
ENSG00000159212	*CLIC6*	4.74E-21	1.064	0.157	0.546
ENSG00000165959	*CLMN*	2.29E-10	1.448	0.354	0.634
ENSG00000149970	*CNKSR2*	3.19E-04	-1.114	-0.029	-0.288
ENSG00000153721	*CNKSR3*	1.78E-17	-1.189	-0.300	-0.547
ENSG00000103647	*CORO2B*	3.84E-07	-1.076	-0.417	-0.594
ENSG00000047457	*CP*	3.73E-35	2.279	0.759	1.220
ENSG00000177685	*CRACR2B*	2.73E-11	-1.369	-0.021	-0.315
ENSG00000213145	*CRIP1*	2.06E-19	-1.028	-0.215	-0.353
ENSG00000121005	*CRISPLD1*	7.42E-26	-1.716	-0.598	-1.002
ENSG00000103196	*CRISPLD2*	5.89E-08	1.684	0.015	0.326
ENSG00000226321	*CROCC2*	8.83E-03	1.235	0.000	0.145
ENSG00000095713	*CRTAC1*	2.18E-13	1.499	0.045	0.664
ENSG00000109943	*CRTAM*	2.23E-03	1.835	0.010	0.113
ENSG00000172346	*CSDC2*	6.74E-05	-1.402	-0.053	-0.553
ENSG00000178662	*CSRNP3*	1.80E-04	-2.323	-0.027	-1.496
ENSG00000118523	*CTGF*	1.27E-14	-1.700	-0.413	-0.908
ENSG00000135047	*CTSL*	1.61E-06	1.024	0.084	0.716
ENSG00000163131	*CTSS*	7.87E-39	1.910	0.366	0.803
ENSG00000006210	*CX3CL1*	2.52E-05	1.445	0.008	0.112
ENSG00000163739	*CXCL1*	1.92E-225	7.081	3.981	4.993
ENSG00000081041	*CXCL2*	5.60E-74	5.839	2.616	3.757
ENSG00000163734	*CXCL3*	4.31E-53	5.404	2.064	3.190
ENSG00000163735	*CXCL5*	2.00E-18	4.569	0.023	2.300

ENSG00000124875	*CXCL6*	1.63E-54	5.656	2.821	3.790
ENSG00000169429	*CXCL8*	2.09E-32	5.969	0.035	2.672
ENSG00000121966	*CXCR4*	3.15E-03	4.545	0.007	0.042
ENSG00000137869	*CYP19A1*	1.41E-02	1.259	0.012	0.161
ENSG00000019186	*CYP24A1*	1.29E-14	4.175	1.879	2.462
ENSG00000146233	*CYP39A1*	1.20E-08	-1.949	-0.535	-0.991
ENSG00000186377	*CYP4X1*	3.75E-04	-1.046	-0.027	-0.310
ENSG00000172817	*CYP7B1*	2.17E-10	1.557	0.501	0.659
ENSG00000205795	*CYS1*	3.28E-13	-1.851	-0.389	-0.888
ENSG00000100592	*DAAM1*	2.96E-20	1.149	0.175	0.420
ENSG00000146122	*DAAM2*	1.98E-07	-1.014	-0.089	-0.593
ENSG00000163331	*DAPL1*	1.26E-06	-2.523	-0.015	-1.630
ENSG00000003249	*DBNDD1*	1.76E-05	-1.215	-0.034	-0.648
ENSG00000105516	*DBP*	2.01E-06	-1.056	-0.053	-0.330
ENSG00000133083	*DCLK1*	7.01E-03	-1.515	-0.018	-0.070
ENSG00000163357	*DCST1*	3.81E-02	-1.075	-0.009	-0.083
ENSG00000153904	*DDAH1*	9.92E-19	-1.398	-0.400	-0.813
ENSG00000168209	*DDIT4*	4.35E-05	1.702	0.018	0.355
ENSG00000145358	*DDIT4L*	5.91E-04	1.253	0.012	0.162
ENSG00000164825	*DEFB1*	3.61E-03	1.231	0.021	0.088
ENSG00000165507	*DEPP1*	3.82E-10	1.926	0.046	0.972
ENSG00000155792	*DEPTOR*	1.63E-15	-2.207	-0.083	-0.908
ENSG00000062282	*DGAT2*	9.04E-12	1.749	0.630	1.305
ENSG00000077044	*DGKD*	1.81E-17	1.152	0.326	0.655
ENSG00000197406	*DIO3*	9.87E-03	2.942	0.008	0.049
ENSG00000140323	*DISP2*	7.79E-04	-1.275	-0.017	-0.984
ENSG00000132837	*DMGDH*	4.38E-07	-1.143	-0.032	-0.200
ENSG00000115423	*DNAH6*	4.98E-02	-1.167	-0.005	-0.103
ENSG00000187957	*DNER*	2.10E-08	1.899	0.014	0.134
ENSG00000149927	*DOC2A*	1.23E-02	-1.316	-0.024	-0.276
ENSG00000128512	*DOCK4*	9.31E-14	1.097	0.049	0.173
ENSG00000136048	*DRAM1*	1.54E-13	1.369	0.399	0.733
ENSG00000136982	*DSCC1*	5.17E-07	-1.138	-0.051	-0.384
ENSG00000134769	*DTNA*	7.01E-06	1.080	0.001	0.265
ENSG00000120129	*DUSP1*	3.91E-16	1.671	0.317	0.870
ENSG00000138166	*DUSP5*	6.11E-12	1.098	0.071	0.579
ENSG00000139318	*DUSP6*	5.52E-17	1.719	0.501	0.919
ENSG00000184545	*DUSP8*	6.49E-21	1.645	0.630	0.957
ENSG00000158560	*DYNC1I1*	2.02E-11	-1.193	-0.150	-0.651
ENSG00000122547	*EEPD1*	1.16E-07	-1.108	-0.292	-0.355

ENSG00000163576	*EFHB*	7.43E-03	-1.231	-0.025	-0.209
ENSG00000115468	*EFHD1*	4.00E-17	-1.699	-0.257	-0.857
ENSG00000129521	*EGLN3*	2.00E-05	1.583	0.022	0.383
ENSG00000013016	*EHD3*	1.27E-05	-1.025	-0.054	-0.676
ENSG00000163435	*ELF3*	4.95E-12	3.004	0.973	1.601
ENSG00000128886	*ELL3*	9.64E-05	-1.023	-0.042	-0.688
ENSG00000197977	*ELOVL2*	1.21E-10	2.657	1.254	2.106
ENSG00000164181	*ELOVL7*	1.08E-03	1.891	0.006	0.054
ENSG00000168913	*ENHO*	1.13E-02	-2.315	-0.003	-0.034
ENSG00000198018	*ENTPD7*	7.13E-20	1.620	0.290	0.682
ENSG00000188833	*ENTPD8*	2.37E-02	-2.824	-0.003	-0.051
ENSG00000082397	*EPB41L3*	7.16E-18	1.390	0.014	0.118
ENSG00000044524	*EPHA3*	3.68E-04	-1.334	-0.028	-0.795
ENSG00000145242	*EPHA5*	3.96E-02	-1.025	-0.009	-0.059
ENSG00000182580	*EPHB3*	1.21E-13	-1.359	-0.224	-0.607
ENSG00000120915	*EPHX2*	6.41E-08	-1.096	-0.242	-0.595
ENSG00000124882	*EREG*	1.59E-02	1.064	0.014	0.172
ENSG00000178752	*ERFE*	8.48E-32	2.633	0.878	1.763
ENSG00000178607	*ERN1*	2.85E-15	1.294	0.067	0.542
ENSG00000116285	*ERRFI1*	7.96E-08	1.221	0.039	0.484
ENSG00000164283	*ESM1*	5.96E-07	1.759	0.037	0.343
ENSG00000157557	*ETS2*	4.64E-13	1.180	0.136	0.569
ENSG00000139083	*ETV6*	9.18E-21	1.194	0.298	0.600
ENSG00000158008	*EXTL1*	4.27E-02	-1.109	-0.016	-0.081
ENSG00000181104	*F2R*	2.26E-16	-1.916	-0.065	-0.713
ENSG00000198734	*F5*	9.27E-04	1.236	0.016	0.368
ENSG00000121769	*FABP3*	1.08E-20	-1.331	-0.307	-0.499
ENSG00000170323	*FABP4*	1.48E-03	-1.263	-0.011	-0.320
ENSG00000169122	*FAM110B*	7.92E-09	1.083	0.069	0.561
ENSG00000135842	*FAM129A*	1.32E-18	-1.874	-0.619	-1.150
ENSG00000179083	*FAM133A*	1.28E-04	-1.181	-0.034	-0.603
ENSG00000109794	*FAM149A*	2.06E-06	-1.280	-0.032	-0.607
ENSG00000154319	*FAM167A*	7.33E-08	1.511	0.046	0.734
ENSG00000185442	*FAM174B*	6.57E-10	1.186	0.113	0.624
ENSG00000197520	*FAM177B*	4.55E-28	4.310	2.489	3.184
ENSG00000108950	*FAM20A*	1.30E-26	2.764	1.197	1.720
ENSG00000177706	*FAM20C*	6.92E-20	1.619	0.427	0.966
ENSG00000185614	*FAM212A*	4.71E-07	-1.540	-0.060	-0.557
ENSG00000157470	*FAM81A*	4.30E-02	-1.023	-0.013	-0.067
ENSG00000162981	*FAM84A*	1.02E-04	-1.271	-0.026	-0.546

ENSG00000165323	*FAT3*	7.84E-09	-1.293	-0.090	-0.837
ENSG00000170271	*FAXDC2*	1.38E-09	-1.342	-0.350	-0.796
ENSG00000165140	*FBP1*	9.42E-12	-1.987	-0.069	-0.856
ENSG00000141665	*FBXO15*	3.04E-03	-1.709	-0.018	-0.176
ENSG00000162894	*FCMR*	1.11E-04	1.898	0.001	0.061
ENSG00000113578	*FGF1*	8.21E-06	1.410	0.013	0.200
ENSG00000070193	*FGF10*	4.31E-24	2.227	0.697	1.249
ENSG00000102466	*FGF14*	7.16E-13	-1.089	-0.236	-0.500
ENSG00000140285	*FGF7*	4.26E-31	1.365	0.505	0.888
ENSG00000137460	*FHDC1*	1.28E-04	1.071	0.016	0.131
ENSG00000022267	*FHL1*	2.13E-21	-1.263	-0.371	-0.648
ENSG00000118407	*FILIP1*	8.06E-17	2.964	0.021	0.363
ENSG00000204131	*FLJ44635*	1.20E-02	-1.523	-0.019	-0.179
ENSG00000075420	*FNDC3B*	1.29E-29	1.237	0.369	0.613
ENSG00000143107	*FNDC7*	2.80E-02	-1.636	-0.015	-0.035
ENSG00000103241	*FOXF1*	7.08E-04	2.075	0.024	1.855
ENSG00000114861	*FOXP1*	3.28E-25	1.069	0.185	0.449
ENSG00000165694	*FRMD7*	1.32E-02	-1.121	-0.026	-0.305
ENSG00000167996	*FTH1*	5.18E-31	1.410	0.322	0.757
ENSG00000010810	*FYN*	4.87E-31	1.242	0.203	0.555
ENSG00000180340	*FZD2*	7.41E-08	-1.184	-0.019	-0.474
ENSG00000123689	*G0S2*	3.45E-30	4.313	1.375	2.400
ENSG00000139112	*GABARAPL1*	4.83E-19	1.177	0.355	0.681
ENSG00000143891	*GALM*	7.21E-09	-1.350	-0.081	-0.552
ENSG00000131386	*GALNT15*	3.92E-28	2.351	0.886	1.441
ENSG00000131979	*GCH1*	1.11E-47	3.628	0.903	1.758
ENSG00000130513	*GDF15*	2.39E-07	1.137	0.528	0.882
ENSG00000125965	*GDF5*	3.78E-19	-1.339	-0.349	-0.615
ENSG00000156466	*GDF6*	3.71E-12	1.012	0.095	0.398
ENSG00000143869	*GDF7*	2.40E-05	-1.355	-0.009	-0.583
ENSG00000158555	*GDPD5*	4.68E-08	-1.045	-0.079	-0.435
ENSG00000131459	*GFPT2*	7.90E-36	1.887	0.514	0.997
ENSG00000168546	*GFRA2*	1.59E-02	1.679	0.004	0.066
ENSG00000179855	*GIPC3*	3.99E-02	-1.063	-0.005	-0.115
ENSG00000198814	*GK*	2.40E-04	1.148	0.035	0.274
ENSG00000074047	*GLI2*	8.46E-09	-1.453	-0.045	-0.931
ENSG00000107249	*GLIS3*	4.98E-18	1.345	0.207	0.594
ENSG00000173221	*GLRX*	1.29E-19	1.729	0.382	0.950
ENSG00000156689	*GLYATL2*	8.77E-03	-1.236	-0.007	-0.217
ENSG00000112312	*GMNN*	2.57E-07	1.101	0.084	0.579

ENSG00000186469	*GNG2*	7.01E-08	-1.149	-0.041	-0.661
ENSG00000176533	*GNG7*	6.70E-05	-1.317	-0.028	-0.213
ENSG00000155265	*GOLGA7B*	6.67E-05	1.299	0.013	0.247
ENSG00000164850	*GPER1*	1.47E-12	-1.042	-0.225	-0.465
ENSG00000112293	*GPLD1*	4.15E-12	-1.010	-0.195	-0.821
ENSG00000180998	*GPR137C*	1.62E-02	-1.281	-0.012	-0.156
ENSG00000158292	*GPR153*	3.62E-15	-1.223	-0.279	-0.611
ENSG00000169508	*GPR183*	2.41E-07	2.841	0.018	0.947
ENSG00000170075	*GPR37L1*	2.68E-09	3.043	0.010	0.106
ENSG00000181656	*GPR88*	2.01E-03	1.175	0.005	0.205
ENSG00000013588	*GPRC5A*	1.99E-19	1.863	0.541	1.080
ENSG00000115290	*GRB14*	4.83E-06	1.441	-0.003	0.094
ENSG00000155511	*GRIA1*	1.73E-08	-1.862	-0.527	-1.065
ENSG00000147697	*GSDMC*	8.53E-03	1.049	0.005	-0.034
ENSG00000169181	*GSG1L*	1.70E-02	-3.352	-0.004	-0.053
ENSG00000134201	*GSTM5*	8.93E-09	-1.463	-0.463	-0.688
ENSG00000164116	*GUCY1A1*	8.76E-06	-1.553	-0.402	-0.745
ENSG00000061918	*GUCY1B1*	3.26E-06	-1.210	-0.022	-0.388
ENSG00000179240	*GVQW3*	7.24E-06	-1.230	-0.334	-0.821
ENSG00000162882	*HAAO*	5.59E-05	-1.156	-0.067	-0.161
ENSG00000188921	*HACD4*	8.18E-21	-1.165	-0.286	-0.705
ENSG00000170961	*HAS2*	1.39E-07	1.691	0.046	0.944
ENSG00000135077	*HAVCR2*	2.96E-04	-1.034	-0.002	-0.301
ENSG00000103145	*HCFC1R1*	9.27E-11	-1.200	-0.343	-0.450
ENSG00000002746	*HECW1*	4.64E-09	-1.226	-0.060	-0.548
ENSG00000135547	*HEY2*	6.04E-05	-1.301	-0.024	-0.563
ENSG00000177374	*HIC1*	7.88E-10	-1.406	-0.061	-0.499
ENSG00000100644	*HIF1A*	1.86E-12	1.171	0.359	0.662
ENSG00000135245	*HILPDA*	8.64E-05	2.547	0.011	0.142
ENSG00000184678	*HIST2H2BE*	4.19E-13	1.125	0.120	0.496
ENSG00000095951	*HIVEP1*	2.00E-18	1.139	0.089	0.336
ENSG00000127124	*HIVEP3*	2.10E-21	1.695	0.575	0.701
ENSG00000159399	*HK2*	1.80E-06	1.444	0.030	0.614
ENSG00000241935	*HOGA1*	5.17E-07	-1.599	0.002	-0.340
ENSG00000196196	*HRCT1*	7.77E-03	-1.700	-0.014	-0.124
ENSG00000117594	*HSD11B1*	4.10E-40	4.420	1.662	2.628
ENSG00000004776	*HSPB6*	1.62E-21	-1.112	-0.435	-0.533
ENSG00000173641	*HSPB7*	1.39E-25	-1.750	-0.719	-1.026
ENSG00000102468	*HTR2A*	6.79E-15	3.417	0.034	1.655
ENSG00000169495	*HTRA4*	4.30E-04	1.547	0.015	0.212

ENSG00000090339	*ICAM1*	7.71E-13	2.080	0.027	0.781
ENSG00000115738	*ID2*	1.16E-26	2.417	0.885	1.336
ENSG00000137331	*IER3*	3.41E-02	1.358	0.011	0.066
ENSG00000185885	*IFITM1*	7.17E-03	1.635	0.014	0.147
ENSG00000142089	*IFITM3*	7.08E-12	1.317	0.359	0.832
ENSG00000017427	*IGF1*	7.44E-11	3.407	0.025	1.588
ENSG00000141753	*IGFBP4*	3.85E-30	1.134	0.232	0.684
ENSG00000164136	*IL15*	3.96E-04	1.093	0.013	0.163
ENSG00000134470	*IL15RA*	9.58E-05	1.052	0.043	0.467
ENSG00000115604	*IL18R1*	4.15E-10	1.238	0.452	0.585
ENSG00000136689	*IL1RN*	2.15E-02	1.394	0.009	0.173
ENSG00000162892	*IL24*	5.89E-05	3.224	0.007	0.037
ENSG00000104998	*IL27RA*	6.73E-04	-1.233	-0.015	-0.188
ENSG00000164509	*IL31RA*	3.25E-12	-1.511	-0.079	-0.912
ENSG00000136696	*IL36B*	4.15E-03	1.027	0.032	0.830
ENSG00000136695	*IL36RN*	3.73E-02	1.626	0.005	0.073
ENSG00000104951	*IL4I1*	7.24E-07	2.266	0.018	0.378
ENSG00000136244	*IL6*	6.73E-32	7.861	0.023	3.915
ENSG00000168685	*IL7R*	8.14E-06	1.089	0.033	0.196
ENSG00000122641	*INHBA*	2.87E-05	1.408	0.019	0.282
ENSG00000188487	*INSC*	3.39E-05	-1.577	-0.048	-1.012
ENSG00000186480	*INSIG1*	2.70E-16	1.471	0.062	0.597
ENSG00000125629	*INSIG2*	4.86E-36	1.206	0.204	0.431
ENSG00000074706	*IPCEF1*	1.22E-03	-1.053	-0.006	-1.443
ENSG00000151151	*IPMK*	7.23E-09	1.161	0.062	0.505
ENSG00000120645	*IQSEC3*	2.90E-03	-1.067	-0.013	-0.188
ENSG00000134070	*IRAK2*	2.59E-27	1.912	0.681	1.117
ENSG00000090376	*IRAK3*	8.60E-17	1.124	0.040	0.266
ENSG00000137265	*IRF4*	1.01E-02	1.761	0.010	0.125
ENSG00000159387	*IRX6*	2.49E-05	-1.550	-0.046	-0.602
ENSG00000101230	*ISM1*	8.74E-05	1.006	0.029	0.330
ENSG00000005961	*ITGA2B*	7.71E-03	-2.161	-0.017	-0.105
ENSG00000115232	*ITGA4*	8.51E-10	-1.026	-0.287	-0.634
ENSG00000105855	*ITGB8*	2.11E-05	1.331	0.023	0.261
ENSG00000150995	*ITPR1*	1.03E-09	1.302	0.047	0.458
ENSG00000148841	*ITPRIP*	3.90E-13	1.375	0.076	0.778
ENSG00000096968	*JAK2*	1.26E-27	1.105	0.115	0.354
ENSG00000188385	*JAKMIP3*	1.32E-04	-1.235	-0.031	-0.255
ENSG00000171223	*JUNB*	3.47E-17	1.836	0.560	1.072
ENSG00000158445	*KCNB1*	6.22E-07	-1.892	-0.031	-1.148

ENSG00000102057	*KCND1*	2.43E-02	-1.019	-0.012	-0.167
ENSG00000152049	*KCNE4*	4.27E-25	1.293	0.509	0.916
ENSG00000143473	*KCNH1*	1.01E-14	1.594	0.047	0.791
ENSG0000055118	*KCNH2*	1.56E-04	-1.158	-0.060	-0.779
ENSG00000157551	*KCNJ15*	2.40E-10	1.409	0.058	0.992
ENSG00000171303	*KCNK3*	6.73E-08	4.271	0.013	3.340
ENSG00000104783	*KCNN4*	1.69E-07	-1.192	-0.037	-0.500
ENSG00000184156	*KCNQ3*	2.03E-21	1.923	0.667	1.085
ENSG00000156486	*KCNS2*	1.06E-02	-1.625	0.002	-0.084
ENSG00000170745	*KCNS3*	2.64E-03	-1.334	-0.011	-0.139
ENSG00000180332	*KCTD4*	1.56E-10	-1.845	-0.057	-0.703
ENSG00000138030	*KHK*	6.01E-03	-1.226	-0.019	-0.067
ENSG00000109265	*KIAA1211*	4.58E-05	-1.109	-0.054	-0.664
ENSG00000162849	*KIF26B*	2.06E-15	-2.017	-0.651	-0.986
ENSG00000155980	*KIF5A*	2.24E-02	-1.404	-0.020	-0.091
ENSG00000157404	*KIT*	3.05E-06	-1.251	-0.710	-0.979
ENSG00000155090	*KLF10*	8.15E-24	1.199	0.390	0.655
ENSG00000171872	*KLF17*	2.34E-08	2.206	0.023	1.025
ENSG00000119138	*KLF9*	2.85E-11	1.064	0.326	0.533
ENSG00000162755	*KLHDC9*	1.02E-04	-1.346	-0.032	-0.303
ENSG00000171345	*KRT19*	1.95E-03	-1.457	-0.023	-0.806
ENSG00000221852	*KRTAP1-5*	2.88E-08	-1.114	-0.311	-0.316
ENSG00000174611	*KY*	2.53E-11	-1.899	-0.774	-0.936
ENSG00000115919	*KYNU*	1.25E-18	3.450	1.416	2.012
ENSG00000101680	*LAMA1*	3.38E-07	1.001	0.156	0.542
ENSG00000129988	*LBP*	1.14E-17	3.270	0.022	1.339
ENSG00000148346	*LCN2*	2.87E-05	2.773	0.020	1.590
ENSG00000136167	*LCP1*	2.22E-02	-1.477	-0.016	-0.053
ENSG00000169744	*LDB2*	1.17E-15	-1.817	-0.449	-0.950
ENSG00000134333	*LDHA*	5.52E-17	1.237	0.421	0.718
ENSG00000166816	*LDHD*	1.08E-07	-1.319	-0.489	-0.798
ENSG00000168675	*LDLRAD4*	1.26E-15	-1.662	-0.704	-1.117
ENSG00000106003	*LFNG*	3.00E-05	-1.194	-0.052	-0.639
ENSG00000153012	*LGI2*	3.69E-08	-1.814	-0.051	-0.935
ENSG00000139292	*LGR5*	3.59E-07	-1.049	-0.295	-0.585
ENSG00000128342	*LIF*	1.03E-07	2.250	0.011	0.180
ENSG00000072163	*LIMS2*	1.03E-11	-1.317	-0.261	-0.608
ENSG00000236882	*LINC01554*	5.61E-06	2.151	0.017	0.194
ENSG00000169783	*LINGO1*	6.64E-05	-1.357	-0.018	-0.699
ENSG00000136153	*LMO7*	3.43E-15	-1.261	-0.189	-0.647

ENSG00000163431	*LMOD1*	2.98E-06	-1.512	-0.040	-0.702
ENSG00000103485	*LOC105369247*	1.49E-03	-2.095	-0.002	-0.063
ENSG00000173535	*LOC254896*	8.92E-04	-1.037	-0.041	-0.189
ENSG00000165379	*LRFN5*	1.30E-02	-1.206	-0.021	-0.354
ENSG00000128606	*LRRC17*	2.49E-09	-2.721	-0.037	-1.610
ENSG00000172731	*LRRC20*	3.28E-08	-1.577	-0.047	-0.690
ENSG00000128594	*LRRC4*	2.20E-06	-2.034	-0.036	-1.102
ENSG00000146006	*LRRTM2*	1.12E-07	-1.450	-0.473	-0.591
ENSG00000130592	*LSP1*	2.67E-14	-1.158	-0.263	-0.473
ENSG00000172264	*MACROD2*	1.49E-05	-1.375	-0.038	-0.533
ENSG00000185022	*MAFF*	2.99E-31	1.474	0.635	0.943
ENSG00000144063	*MALL*	9.59E-08	1.106	0.098	0.610
ENSG00000165072	*MAMDC2*	7.68E-35	-2.104	-0.570	-1.126
ENSG00000196782	*MAML3*	5.86E-04	-1.231	-1.017	-1.138
ENSG00000013619	*MAMLD1*	2.35E-20	-1.770	-0.748	-1.382
ENSG00000176909	*MAMSTR*	3.04E-04	-1.423	-0.035	-0.290
ENSG00000069535	*MAOB*	1.16E-15	1.803	0.052	0.622
ENSG00000197769	*MAP1LC3C*	5.86E-13	-2.132	-1.052	-1.715
ENSG00000108984	*MAP2K6*	5.48E-08	-2.502	-0.827	-1.438
ENSG00000107968	*MAP3K8*	1.85E-188	2.425	1.551	1.929
ENSG00000186868	*MAPT*	1.17E-02	-1.235	-0.019	-0.713
ENSG00000186205	*MARC1*	8.69E-05	-1.215	-0.629	-0.541
ENSG00000173926	*MARCH3*	1.68E-15	2.353	0.014	0.655
ENSG00000144583	*MARCH4*	1.52E-03	-1.606	-0.018	-0.704
ENSG00000172197	*MBOAT1*	1.29E-10	-1.210	-0.092	-0.868
ENSG00000140563	*MCTP2*	1.30E-06	1.065	0.021	0.217
ENSG00000102802	*MEDAG*	4.18E-37	2.175	0.820	1.351
ENSG00000106511	*MEOX2*	2.16E-21	-1.828	-0.423	-1.094
ENSG00000188095	*MESP2*	3.57E-02	-1.320	-0.012	-0.135
ENSG00000170439	*METTL7B*	1.78E-07	-2.638	-0.029	-1.219
ENSG00000168389	*MFSD2A*	7.40E-09	2.118	0.036	1.157
ENSG00000174514	*MFSD4A*	2.24E-02	-1.057	-0.017	-0.783
ENSG00000108960	*MMD*	1.86E-10	1.187	0.689	0.761
ENSG00000196549	*MME*	1.59E-10	1.361	0.047	0.474
ENSG00000196611	*MMP1*	1.83E-39	6.069	1.925	3.486
ENSG00000166670	*MMP10*	1.15E-11	2.147	0.014	0.419
ENSG00000137745	*MMP13*	2.54E-35	3.161	0.037	1.131
ENSG00000149968	*MMP3*	7.44E-59	4.451	0.798	1.889
ENSG00000120162	*MOB3B*	2.94E-04	-0.715	-1.202	-0.217
ENSG00000186732	*MPPED1*	5.45E-03	-1.342	-0.009	-0.945

ENSG00000066382	*MPPED2*	4.07E-05	-1.193	-0.040	-0.469
ENSG00000156968	*MPV17L*	1.06E-07	-1.718	-0.037	-0.998
ENSG00000135324	*MRAP2*	2.12E-15	-1.915	-0.644	-1.070
ENSG00000184350	*MRGPRE*	2.08E-02	-1.190	-0.007	-0.170
ENSG00000072952	*MRVI1*	3.51E-15	-1.650	-0.470	-1.144
ENSG00000178860	*MSC*	8.42E-07	1.032	0.033	0.277
ENSG00000205362	*MT1A*	1.09E-02	1.320	0.017	0.057
ENSG00000169715	*MT1E*	3.11E-14	1.362	0.488	0.934
ENSG00000198417	*MT1F*	2.43E-17	2.308	0.049	1.192
ENSG00000125144	*MT1G*	1.83E-09	3.682	0.024	2.092
ENSG00000205364	*MT1M*	8.46E-08	1.277	0.041	0.518
ENSG00000187193	*MT1X*	1.12E-14	2.105	0.681	1.340
ENSG00000125148	*MT2A*	4.18E-47	2.423	0.980	1.507
ENSG00000242114	*MTFP1*	5.72E-04	1.613	0.027	0.818
ENSG00000185499	*MUC1*	1.09E-06	1.112	0.348	0.610
ENSG00000133055	*MYBPH*	2.52E-10	1.754	0.544	1.003
ENSG00000136449	*MYCBPAP*	2.90E-04	-1.234	-0.052	-0.666
ENSG00000065534	*MYLK*	5.17E-08	-1.189	-0.073	-0.790
ENSG00000128833	*MYO5C*	5.30E-03	-1.058	-0.023	-0.329
ENSG00000034971	*MYOC*	2.57E-14	-1.238	-0.441	-0.734
ENSG00000105835	*NAMPT*	9.42E-20	3.291	0.037	1.523
ENSG00000186462	*NAP1L2*	2.21E-05	-1.184	-0.475	-0.503
ENSG00000186310	*NAP1L3*	4.37E-07	-1.375	-0.038	-0.687
ENSG00000156006	*NAT2*	3.67E-03	-1.105	-0.044	-0.178
ENSG00000067798	*NAV3*	8.35E-05	-1.072	-0.035	-0.567
ENSG00000160796	*NBEAL2*	3.67E-05	-1.076	-0.074	-0.788
ENSG00000104490	*NCALD*	1.84E-05	-1.977	-0.028	-0.519
ENSG00000154654	*NCAM2*	3.37E-11	2.096	0.034	0.896
ENSG00000124479	*NDP*	7.29E-06	1.865	0.014	0.173
ENSG00000104419	*NDRG1*	6.40E-16	1.720	0.073	0.803
ENSG00000165795	*NDRG2*	7.29E-06	-1.302	-0.021	-0.584
ENSG00000165030	*NFIL3*	3.99E-21	1.858	0.438	1.058
ENSG00000077150	*NFKB2*	2.86E-16	1.108	0.037	0.339
ENSG00000100906	*NFKBIA*	1.63E-67	2.447	0.889	1.343
ENSG00000144802	*NFKBIZ*	2.09E-246	3.596	2.648	2.975
ENSG00000257108	*NHLRC4*	2.57E-02	-1.066	-0.019	-0.085
ENSG00000204131	*NHSL2*	1.20E-02	-1.523	-0.019	-0.179
ENSG00000165028	*NIPSNAP3B*	2.75E-06	-1.095	-0.336	-0.463
ENSG00000140807	*NKD1*	4.11E-06	-1.014	-0.043	-0.825
ENSG00000167034	*NKX3-1*	8.92E-18	1.420	0.426	0.511

ENSG00000182261	*NLRP10*	4.17E-05	-2.052	-0.020	-0.847
ENSG00000166741	*NNMT*	1.43E-42	2.245	0.990	1.587
ENSG00000151014	*NOCT*	4.89E-08	1.256	0.031	0.548
ENSG00000167207	*NOD2*	2.53E-24	4.202	0.024	2.032
ENSG00000007171	*NOS2*	7.49E-36	7.417	4.309	5.152
ENSG00000163072	*NOSTRIN*	4.57E-02	-1.134	-0.005	-0.002
ENSG00000136999	*NOV*	4.23E-12	-1.249	-0.062	-0.419
ENSG00000139910	*NOVA1*	5.31E-09	1.037	0.118	0.593
ENSG00000171246	*NPTX1*	1.63E-10	-1.653	-0.607	-0.832
ENSG00000144852	*NR1I2*	3.85E-02	-1.893	-0.012	-0.032
ENSG00000151623	*NR3C2*	7.96E-16	-1.322	-0.309	-0.643
ENSG00000153234	*NR4A2*	2.87E-12	1.755	0.068	0.798
ENSG00000119508	*NR4A3*	2.63E-29	2.536	0.865	1.557
ENSG00000134986	*NREP*	1.58E-15	-1.584	-0.520	-0.995
ENSG00000118257	*NRP2*	9.77E-17	1.388	0.160	0.660
ENSG00000065320	*NTN1*	1.89E-13	1.528	0.386	0.982
ENSG00000148053	*NTRK2*	4.29E-12	-1.044	-0.335	-0.722
ENSG00000186364	*NUDT17*	2.27E-05	-1.084	-0.039	-0.779
ENSG00000182575	*NXPH3*	6.62E-09	-1.428	-0.416	-0.977
ENSG00000205978	*NYNRIN*	3.89E-08	-1.090	-0.192	-0.597
ENSG00000154358	*OBSCN*	1.43E-18	-1.093	-0.384	-0.605
ENSG00000177989	*ODF3B*	2.35E-06	1.401	0.043	0.698
ENSG00000105088	*OLFM2*	8.91E-14	-1.224	-0.255	-0.441
ENSG00000183801	*OLFML1*	2.80E-04	-1.793	-0.035	-0.503
ENSG00000162745	*OLFML2B*	1.45E-13	-1.123	-0.243	-0.460
ENSG00000127083	*OMD*	3.59E-10	-1.017	-0.102	-0.606
ENSG00000183715	*OPCML*	1.39E-02	-1.587	-0.018	-0.102
ENSG00000125510	*OPRL1*	4.77E-05	-1.003	-0.040	-0.518
ENSG00000070882	*OSBPL3*	2.33E-18	-1.226	-0.353	-0.711
ENSG00000006025	*OSBPL7*	2.70E-08	-1.162	-0.125	-0.601
ENSG00000145623	*OSMR*	2.37E-40	2.286	0.869	1.354
ENSG00000180914	*OXTR*	1.12E-03	-1.421	-0.018	-0.105
ENSG00000099957	*P2RX6*	1.85E-06	-1.437	-0.037	-0.787
ENSG00000076641	*PAG1*	2.47E-06	1.096	0.033	0.222
ENSG00000243444	*PALM2*	6.84E-08	1.626	0.064	1.128
ENSG00000157654	*PALM2-AKAP2*	3.63E-03	2.753	0.006	0.068
ENSG00000099260	*PALMD*	8.47E-22	1.838	0.610	1.063
ENSG00000162073	*PAQR4*	1.16E-04	-1.095	-0.013	-0.213
ENSG00000102981	*PARD6A*	3.84E-04	-1.009	-0.055	-0.703
ENSG00000173599	*PC*	1.16E-08	1.102	0.094	0.595

ENSG00000183570	*PCBP3*	2.24E-09	1.408	0.017	0.330
ENSG00000150275	*PCDH15*	9.74E-03	1.621	0.010	0.034
ENSG00000189184	*PCDH18*	1.18E-07	-1.175	-0.370	-0.875
ENSG00000124253	*PCK1*	3.93E-04	4.552	0.006	0.072
ENSG00000175426	*PCSK1*	3.51E-17	1.822	0.362	0.911
ENSG00000172572	*PDE3A*	1.27E-09	1.132	0.010	0.083
ENSG00000184588	*PDE4B*	1.22E-61	2.876	1.289	1.816
ENSG00000113448	*PDE4D*	1.21E-26	2.308	0.456	1.006
ENSG00000138735	*PDE5A*	7.21E-12	-2.086	-0.605	-1.322
ENSG00000170962	*PDGFD*	1.10E-10	-1.129	-0.119	-0.568
ENSG00000152256	*PDK1*	1.17E-15	1.455	0.228	0.679
ENSG00000162493	*PDPN*	2.79E-23	1.576	0.557	1.008
ENSG00000186862	*PDZD7*	6.67E-03	-1.008	-0.018	-0.609
ENSG00000162366	*PDZK1IP1*	4.96E-09	2.736	0.026	1.388
ENSG00000109272	*PF4V1*	1.88E-03	2.203	0.018	1.334
ENSG00000170525	*PFKFB3*	1.36E-09	1.320	0.080	0.711
ENSG00000070087	*PFN2*	4.22E-20	-1.150	-0.289	-0.579
ENSG00000176732	*PFN4*	5.81E-03	-1.269	-0.017	-0.170
ENSG00000119630	*PGF*	5.62E-08	2.104	0.006	0.080
ENSG00000102144	*PGK1*	7.99E-12	1.083	0.245	0.540
ENSG00000153823	*PID1*	4.07E-27	1.845	0.017	0.266
ENSG00000137193	*PIM1*	8.32E-19	1.371	0.588	1.037
ENSG00000198355	*PIM3*	5.24E-13	1.384	0.515	0.885
ENSG00000154217	*PITPNC1*	2.86E-05	1.126	0.027	0.413
ENSG00000166473	*PKD1L2*	6.35E-06	-1.692	-0.017	-1.149
ENSG00000165495	*PKNOX2*	4.56E-37	2.080	0.482	1.166
ENSG00000138193	*PLCE1*	1.01E-16	-1.461	-0.450	-0.944
ENSG00000075651	*PLD1*	7.71E-67	2.041	0.598	1.030
ENSG00000021300	*PLEKHB1*	1.03E-03	-1.057	-0.018	-0.142
ENSG00000171680	*PLEKHG5*	5.12E-08	-1.025	-0.234	-0.379
ENSG00000147872	*PLIN2*	2.19E-03	1.060	0.025	0.435
ENSG00000145632	*PLK2*	1.70E-08	1.122	0.024	0.201
ENSG00000152952	*PLOD2*	2.87E-12	1.370	0.249	0.620
ENSG00000120756	*PLS1*	5.92E-04	-1.200	-0.036	-0.442
ENSG00000188313	*PLSCR1*	3.32E-03	1.213	0.005	0.073
ENSG00000221866	*PLXNA4*	4.69E-09	3.372	1.266	1.980
ENSG00000141682	*PMAIP1*	1.81E-10	1.965	0.979	1.539
ENSG00000182013	*PNMA8A*	6.63E-06	-1.479	-0.030	-0.915
ENSG00000204851	*PNMA8B*	1.14E-04	-1.291	-0.042	-0.446
ENSG00000130653	*PNPLA7*	1.23E-08	-1.385	-0.092	-0.712

ENSG00000146278	*PNRC1*	4.82E-06	1.054	0.025	0.273
ENSG00000028277	*POU2F2*	1.15E-29	2.726	1.458	1.909
ENSG00000109819	*PPARGC1A*	2.83E-03	1.321	0.027	0.644
ENSG00000110841	*PPFIBP1*	2.21E-19	1.046	0.176	0.470
ENSG00000108179	*PPIF*	5.14E-16	1.278	0.395	0.791
ENSG00000156475	*PPP2R2B*	6.70E-11	-2.243	-0.045	-1.067
ENSG00000214140	*PRCD*	1.96E-02	-1.622	-0.013	-0.106
ENSG00000057657	*PRDM1*	2.00E-04	1.887	0.010	0.099
ENSG00000186652	*PRG2*	1.81E-02	1.104	0.032	0.516
ENSG00000139174	*PRICKLE1*	2.64E-10	-1.465	-0.455	-0.998
ENSG00000146143	*PRIM2*	1.14E-08	-1.246	-0.077	-0.616
ENSG00000175785	*PRIMA1*	1.11E-03	-1.537	-0.006	-0.280
ENSG00000005249	*PRKAR2B*	7.90E-23	1.903	0.063	0.994
ENSG00000138669	*PRKG2*	5.05E-04	-1.822	-0.028	-0.314
ENSG00000135406	*PRPH*	1.71E-06	-1.918	-0.037	-0.518
ENSG00000167371	*PRRT2*	1.99E-12	-1.247	-0.409	-0.676
ENSG00000146250	*PRSS35*	1.28E-11	-2.521	-0.046	-1.041
ENSG00000125637	*PSD4*	6.35E-08	1.226	0.065	0.595
ENSG00000117425	*PTCH2*	2.59E-09	-1.434	-0.073	-0.783
ENSG00000148344	*PTGES*	6.11E-14	2.616	0.022	0.466
ENSG00000122420	*PTGFR*	1.91E-45	2.022	0.871	1.315
ENSG00000073756	*PTGS2*	4.17E-12	3.153	0.016	0.546
ENSG00000087494	*PTHLH*	9.10E-07	1.282	0.561	1.080
ENSG00000163629	*PTPN13*	2.79E-14	-1.131	-0.366	-0.767
ENSG00000163661	*PTX3*	4.05E-04	1.148	0.038	0.566
ENSG00000068976	*PYGM*	4.76E-03	-1.517	-0.025	-0.396
ENSG00000103485	*QPRT*	1.49E-03	-2.095	-0.002	-0.063
ENSG00000139832	*RAB20*	2.62E-04	1.252	0.032	0.677
ENSG00000167964	*RAB26*	9.84E-06	-1.505	-0.034	-0.465
ENSG00000041353	*RAB27B*	1.02E-25	2.460	1.060	1.576
ENSG00000172794	*RAB37*	1.75E-04	-1.168	-0.035	-0.530
ENSG00000169750	*RAC3*	5.60E-10	-1.220	-0.057	-0.306
ENSG00000166349	*RAG1*	1.73E-04	-1.221	-0.018	-0.840
ENSG00000164188	*RANBP3L*	2.29E-02	-1.006	0.001	-0.093
ENSG00000108551	*RASD1*	2.39E-10	2.710	0.024	1.313
ENSG00000103710	*RASL12*	1.29E-05	-1.542	-0.038	-0.967
ENSG00000163694	*RBM47*	3.45E-04	1.122	0.037	0.494
ENSG00000159200	*RCAN1*	2.03E-07	1.210	0.024	0.337
ENSG00000172348	*RCAN2*	7.86E-18	-3.059	-0.077	-1.624
ENSG00000167771	*RCOR2*	5.78E-05	-1.537	-0.033	-0.566

ENSG00000104856	*RELB*	2.20E-22	1.716	0.334	0.760
ENSG00000054967	*RELT*	3.67E-06	1.316	0.022	0.982
ENSG00000088320	*REM1*	1.07E-04	-1.040	-0.037	-0.306
ENSG00000174236	*REP15*	1.99E-04	1.104	0.039	0.467
ENSG00000154153	*RETREG1*	1.18E-17	1.586	0.356	0.817
ENSG00000009413	*REV3L*	2.18E-17	1.223	0.154	0.477
ENSG00000178882	*RFLNA*	2.75E-04	-1.472	-0.038	-0.755
ENSG00000131378	*RFTN1*	1.55E-18	-1.043	-0.387	-0.651
ENSG00000143333	*RGS16*	2.21E-13	1.196	0.341	0.625
ENSG00000132554	*RGS22*	1.49E-02	-1.142	-0.024	-0.206
ENSG00000108370	*RGS9*	1.58E-02	-1.429	-0.015	-0.133
ENSG00000072422	*RHOBTB1*	3.65E-17	-1.372	-0.532	-0.866
ENSG00000111913	*RIPOR2*	4.17E-06	1.034	0.020	0.222
ENSG00000042062	*RIPOR3*	6.20E-07	1.573	0.014	0.670
ENSG00000108830	*RND2*	9.52E-04	-1.250	-0.020	-0.157
ENSG00000101695	*RNF125*	1.10E-03	-1.462	-0.019	-0.917
ENSG00000137393	*RNF144B*	2.03E-10	1.068	0.008	0.124
ENSG00000108375	*RNF43*	3.04E-05	-1.388	-0.057	-0.583
ENSG00000185008	*ROBO2*	1.81E-07	-0.969	-0.279	-1.007
ENSG00000169071	*ROR2*	4.34E-06	1.114	0.086	0.511
ENSG00000143365	*RORC*	2.25E-02	-1.433	-0.015	-0.133
ENSG00000156313	*RPGR*	2.81E-09	1.026	0.271	0.686
ENSG00000215853	*RPTN*	1.48E-02	-1.545	-0.016	-0.157
ENSG00000166592	*RRAD*	1.48E-05	1.292	0.033	0.589
ENSG00000146374	*RSPO3*	4.14E-20	1.701	0.633	1.056
ENSG00000179300	*RTL3*	1.32E-04	-1.032	-0.040	-0.438
ENSG00000242732	*RTL5*	3.00E-17	-1.066	-0.425	-0.529
ENSG00000040608	*RTN4R*	4.82E-02	-1.059	0.000	-0.070
ENSG00000186907	*RTN4RL2*	3.21E-09	2.475	0.029	1.593
ENSG00000171509	*RXFP1*	2.70E-03	1.111	0.030	0.109
ENSG00000170989	*S1PR1*	5.56E-11	1.886	0.537	1.197
ENSG00000213694	*S1PR3*	1.23E-05	1.144	0.030	0.454
ENSG00000173432	*SAA1*	2.81E-28	6.628	0.033	4.583
ENSG00000134339	*SAA2*	1.63E-17	6.156	0.030	4.201
ENSG00000177570	*SAMD12*	8.30E-06	-1.894	-0.012	-0.956
ENSG00000203943	*SAMD13*	5.60E-04	-1.538	-0.024	-0.684
ENSG00000164483	*SAMD3*	1.34E-02	-1.185	-0.008	-0.171
ENSG00000203727	*SAMD5*	8.65E-15	-1.299	-0.267	-0.728
ENSG00000123453	*SARDH*	6.98E-05	-1.678	-0.026	-0.550
ENSG00000130066	*SAT1*	1.03E-30	1.594	0.276	0.648

ENSG00000064932	*SBNO2*	7.65E-27	1.791	0.783	1.247
ENSG00000073060	*SCARB1*	3.38E-12	1.087	0.370	0.685
ENSG00000184178	*SCFD2*	2.13E-10	-1.104	-0.409	-0.653
ENSG00000171951	*SCG2*	3.17E-06	1.217	0.428	0.888
ENSG00000161929	*SCIMP*	6.46E-04	-1.006	-0.013	-0.521
ENSG00000006747	*SCIN*	1.97E-06	-1.206	-0.041	-0.542
ENSG00000136531	*SCN2A*	6.29E-03	-1.399	-0.017	-0.340
ENSG00000149575	*SCN2B*	1.97E-08	-1.536	-0.050	-0.568
ENSG00000153253	*SCN3A*	5.28E-03	-1.499	-0.010	-0.838
ENSG00000103184	*SEC14L5*	4.45E-04	-1.255	-0.591	-0.829
ENSG00000120341	*SEC16B*	3.21E-05	-1.076	-0.035	-0.696
ENSG00000174175	*SELP*	3.55E-04	1.944	0.009	0.148
ENSG00000170381	*SEMA3E*	1.31E-14	-1.120	-0.188	-0.596
ENSG00000137872	*SEMA6D*	1.75E-05	1.014	0.018	0.174
ENSG00000108387	*SEPT4*	1.86E-05	-1.468	-0.036	-0.658
ENSG00000135919	*SERPINE2*	4.30E-11	1.381	0.056	0.539
ENSG00000198879	*SFMBT2*	1.84E-08	1.439	0.004	0.195
ENSG00000104332	*SFRP1*	8.13E-11	2.622	0.031	1.204
ENSG00000167037	*SGSM1*	5.88E-04	-1.524	-0.013	-0.112
ENSG00000183918	*SH2D1A*	1.57E-02	-1.499	-0.011	-0.103
ENSG00000169247	*SH3TC2*	4.95E-03	-1.051	-0.020	-0.676
ENSG00000162105	*SHANK2*	1.54E-05	-1.122	-0.057	-0.680
ENSG00000107338	*SHB*	1.68E-17	1.951	0.422	1.116
ENSG00000185634	*SHC4*	7.72E-07	-1.502	-0.477	-0.702
ENSG00000138771	*SHROOM3*	2.40E-13	-1.768	-0.622	-1.255
ENSG00000197046	*SIGLEC15*	4.55E-03	-1.345	-0.019	-0.930
ENSG00000142178	*SIK1*	4.87E-16	2.314	0.038	1.065
ENSG00000198053	*SIRPA*	3.08E-34	1.125	0.116	0.409
ENSG00000101307	*SIRPB1*	1.20E-04	1.119	0.031	0.163
ENSG00000110911	*SLC11A2*	1.21E-34	1.278	0.495	0.731
ENSG00000141469	*SLC14A1*	2.60E-16	-3.065	-1.073	-1.855
ENSG00000112394	*SLC16A10*	9.05E-07	3.011	0.003	0.089
ENSG00000174326	*SLC16A11*	2.40E-02	-1.070	-0.016	0.011
ENSG00000163053	*SLC16A14*	2.86E-06	-1.387	-0.033	-0.592
ENSG00000141526	*SLC16A3*	2.02E-11	1.187	0.253	0.723
ENSG00000108932	*SLC16A6*	4.90E-02	1.236	0.004	0.011
ENSG00000118596	*SLC16A7*	2.84E-12	1.629	0.059	0.591
ENSG00000165449	*SLC16A9*	1.05E-05	-1.593	-0.041	-0.436
ENSG00000146409	*SLC18B1*	1.41E-13	1.132	0.097	0.451
ENSG00000117479	*SLC19A2*	1.26E-12	1.334	0.069	0.654

ENSG00000135917	*SLC19A3*	1.32E-43	3.422	1.244	2.043
ENSG00000112499	*SLC22A2*	7.10E-03	1.944	0.003	0.024
ENSG00000137266	*SLC22A23*	9.23E-20	1.576	0.620	0.964
ENSG00000258708	*SLC25A21-AS1*	6.55E-03	-1.002	-0.031	-0.357
ENSG00000155287	*SLC25A28*	1.62E-21	1.803	0.185	0.818
ENSG00000147454	*SLC25A37*	1.43E-11	1.069	0.040	0.306
ENSG00000091137	*SLC26A4*	3.33E-07	-1.623	-0.046	-0.932
ENSG00000174502	*SLC26A9*	2.87E-05	2.849	0.001	0.067
ENSG00000143554	*SLC27A3*	4.65E-09	-1.113	-0.056	-0.408
ENSG00000117394	*SLC2A1*	2.22E-06	1.119	0.026	0.473
ENSG00000146411	*SLC2A12*	1.70E-09	-1.417	-0.060	-0.882
ENSG00000059804	*SLC2A3*	1.72E-06	1.590	0.033	0.740
ENSG00000142583	*SLC2A5*	1.50E-23	2.401	0.893	1.618
ENSG00000170385	*SLC30A1*	4.90E-27	1.591	0.522	0.818
ENSG00000139209	*SLC38A4*	4.18E-05	-1.631	-0.022	-0.983
ENSG00000104635	*SLC39A14*	3.49E-63	3.314	1.548	2.172
ENSG00000138821	*SLC39A8*	6.20E-40	3.420	0.048	1.223
ENSG00000138449	*SLC40A1*	2.74E-15	-1.888	-0.079	-0.930
ENSG00000167703	*SLC43A2*	6.82E-43	2.283	0.761	1.247
ENSG00000134802	*SLC43A3*	1.38E-27	1.441	0.406	0.840
ENSG00000180638	*SLC47A2*	3.29E-09	-1.426	-0.261	-0.466
ENSG00000131389	*SLC6A6*	5.46E-06	1.261	0.036	0.374
ENSG00000196517	*SLC6A9*	1.99E-06	1.089	0.043	0.550
ENSG00000003989	*SLC7A2*	5.29E-29	3.335	0.804	1.424
ENSG00000103257	*SLC7A5*	4.02E-10	1.743	0.039	0.736
ENSG00000183023	*SLC8A1*	1.00E-17	-1.473	-0.428	-0.946
ENSG00000124107	*SLPI*	4.44E-10	1.593	0.042	0.885
ENSG00000198732	*SMOC1*	2.13E-04	1.385	0.011	0.172
ENSG00000088826	*SMOX*	2.44E-10	1.584	0.449	0.837
ENSG00000124216	*SNAI1*	5.57E-05	1.017	0.057	0.435
ENSG00000065609	*SNAP91*	1.17E-02	-1.169	-0.014	-1.511
ENSG00000023608	*SNAPC1*	1.09E-11	1.102	0.346	0.619
ENSG00000145335	*SNCA*	7.74E-03	-1.663	-0.008	-0.063
ENSG00000064692	*SNCAIP*	1.36E-02	-1.093	-0.030	-0.169
ENSG00000173267	*SNCG*	5.00E-11	-1.374	-0.092	-0.485
ENSG00000184557	*SOCS3*	2.99E-31	1.734	0.544	1.037
ENSG00000112096	*SOD2*	1.02E-44	4.185	1.970	2.777
ENSG00000112096	*SOD2-OT1*	1.02E-44	4.185	1.970	2.777
ENSG00000137642	*SORL1*	2.25E-06	-1.024	-0.244	-0.817
ENSG00000137098	*SPAG8*	1.90E-03	-1.073	-0.029	-0.424

ENSG00000164266	*SPINK1*	2.27E-02	1.480	0.019	1.844
ENSG00000187678	*SPRY4*	1.89E-10	1.449	0.021	0.432
ENSG00000137877	*SPTBN5*	1.88E-03	-1.169	-0.035	-1.017
ENSG00000196542	*SPTSSB*	2.34E-09	-2.019	-0.054	-1.195
ENSG00000122862	*SRGN*	7.70E-23	1.195	0.041	0.298
ENSG00000184343	*SRPK3*	6.40E-05	-1.339	-0.020	-0.545
ENSG00000117155	*SSX2IP*	1.90E-10	-1.174	-0.051	-0.495
ENSG00000144057	*ST6GAL2*	6.48E-03	-1.614	-0.021	-0.095
ENSG00000136840	*ST6GALNAC4*	1.35E-13	1.020	0.371	0.660
ENSG00000141750	*STAC2*	3.87E-08	2.019	0.017	0.660
ENSG00000168610	*STAT3*	5.08E-31	1.279	0.465	0.811
ENSG00000159167	*STC1*	2.29E-40	4.459	1.831	2.826
ENSG00000164647	*STEAP1*	1.18E-45	2.596	0.880	1.521
ENSG00000157214	*STEAP2*	4.31E-24	1.844	0.085	0.719
ENSG00000127954	*STEAP4*	1.34E-12	2.272	0.040	0.895
ENSG00000165209	*STRBP*	7.30E-16	-1.072	-0.271	-0.513
ENSG00000128578	*STRIP2*	2.18E-11	1.818	0.834	1.360
ENSG00000135604	*STX11*	1.05E-03	1.177	0.028	0.541
ENSG00000130540	*SULT4A1*	4.48E-05	1.355	-0.002	0.112
ENSG00000078269	*SYNJ2*	1.68E-23	1.294	0.469	0.747
ENSG00000182253	*SYNM*	1.57E-19	-1.167	-0.318	-0.625
ENSG00000143028	*SYPL2*	2.83E-02	-1.091	-0.006	-0.050
ENSG00000019505	*SYT13*	1.33E-02	-1.629	-0.008	-0.136
ENSG00000137501	*SYTL2*	9.06E-08	-1.076	-0.069	-0.535
ENSG00000165929	*TC2N*	2.56E-09	2.618	0.019	1.394
ENSG00000081059	*TCF7*	2.28E-11	-1.165	-0.069	-0.407
ENSG00000182134	*TDRKH*	1.44E-04	-1.218	-0.027	-0.640
ENSG00000120156	*TEK*	8.87E-08	-1.877	-0.045	-1.082
ENSG00000105825	*TFPI2*	1.65E-07	2.166	0.023	0.842
ENSG00000072274	*TFRC*	3.83E-14	1.188	0.458	0.663
ENSG00000092969	*TGFB2*	9.61E-19	-1.089	-0.409	-0.715
ENSG00000106799	*TGFBR1*	1.02E-22	1.125	0.300	0.561
ENSG00000177426	*TGIF1*	1.38E-11	1.042	0.259	0.596
ENSG00000157150	*TIMP4*	5.58E-07	-1.138	-0.039	-0.482
ENSG00000137462	*TLR2*	4.35E-06	2.375	0.009	0.104
ENSG00000187554	*TLR5*	3.71E-05	-1.362	-0.040	-0.703
ENSG00000166292	*TMEM100*	7.77E-11	2.097	0.771	0.944
ENSG00000183160	*TMEM119*	1.53E-23	-2.257	-0.682	-1.119
ENSG00000166448	*TMEM130*	1.57E-02	-1.677	-0.014	-0.147
ENSG00000157111	*TMEM171*	4.35E-13	-1.651	-0.516	-1.198

ENSG00000172738	*TMEM217*	8.14E-06	1.647	0.025	0.838
ENSG00000196932	*TMEM26*	9.94E-24	-2.393	-0.813	-1.337
ENSG00000126950	*TMEM35A*	1.73E-05	-2.574	-0.020	-0.257
ENSG00000171227	*TMEM37*	2.49E-03	-1.280	-0.033	-0.864
ENSG00000147027	*TMEM47*	1.94E-34	-1.079	-0.395	-0.728
ENSG00000136842	*TMOD1*	7.91E-20	2.336	0.786	1.473
ENSG00000128872	*TMOD2*	2.31E-05	-1.158	-0.042	-0.790
ENSG00000041982	*TNC*	3.92E-14	1.354	0.075	0.616
ENSG00000185215	*TNFAIP2*	1.79E-24	1.668	0.071	0.535
ENSG00000118503	*TNFAIP3*	2.62E-50	1.978	0.262	0.675
ENSG00000123610	*TNFAIP6*	9.68E-38	5.293	1.605	2.813
ENSG00000145779	*TNFAIP8*	6.62E-12	1.202	0.010	0.184
ENSG00000173535	*TNFRSF10C*	8.92E-04	-1.037	-0.041	-0.189
ENSG00000164761	*TNFRSF11B*	2.72E-15	1.711	0.580	0.971
ENSG00000127863	*TNFRSF19*	1.00E-17	-2.323	-0.753	-1.361
ENSG00000028137	*TNFRSF1B*	6.99E-21	2.501	0.464	1.185
ENSG00000049249	*TNFRSF9*	1.70E-04	1.759	0.007	0.062
ENSG00000121858	*TNFSF10*	3.79E-02	1.039	0.010	0.051
ENSG00000120659	*TNFSF11*	5.25E-04	2.614	0.010	0.123
ENSG00000154310	*TNIK*	2.64E-14	1.084	0.219	0.328
ENSG00000131746	*TNS4*	1.18E-05	1.562	0.028	0.695
ENSG00000198846	*TOX*	1.00E-12	-2.133	-0.060	-1.047
ENSG00000078804	*TP53INP2*	3.82E-25	1.166	0.216	0.569
ENSG00000111669	*TPI1*	1.53E-12	1.024	0.260	0.588
ENSG00000056972	*TRAF3IP2*	1.81E-17	1.010	0.325	0.637
ENSG00000124731	*TREM1*	3.71E-09	3.986	0.010	1.826
ENSG00000173334	*TRIB1*	9.21E-19	1.772	0.837	1.302
ENSG00000121060	*TRIM25*	2.96E-06	1.020	0.008	0.157
ENSG00000134253	*TRIM45*	1.69E-07	-1.413	-0.046	-0.880
ENSG00000158022	*TRIM63*	3.45E-02	1.631	0.016	0.133
ENSG00000137672	*TRPC6*	2.99E-06	-1.897	-0.025	-0.760
ENSG00000157514	*TSC22D3*	2.73E-24	-1.014	-0.255	-0.374
ENSG00000099282	*TSPAN15*	1.58E-04	-1.463	-0.032	-0.238
ENSG00000157570	*TSPAN18*	6.47E-07	-1.570	-0.036	-0.681
ENSG00000064201	*TSPAN32*	2.54E-07	-1.578	-0.026	-0.736
ENSG00000122691	*TWIST1*	2.52E-17	1.387	0.456	0.825
ENSG00000025708	*TYMP*	3.81E-11	2.102	0.046	1.054
ENSG00000145040	*UCN2*	5.69E-05	1.270	-0.003	0.000
ENSG00000112715	*VEGFA*	2.62E-08	1.960	0.030	0.796
ENSG00000112299	*VNN1*	1.20E-31	3.323	0.048	1.656

ENSG00000112303	*VNN2*	1.89E-02	1.555	0.003	0.056
ENSG00000093134	*VNN3*	7.11E-26	5.096	0.014	2.715
ENSG00000214376	*VSTM5*	5.62E-04	-2.034	-0.008	-0.722
ENSG00000188730	*VWC2*	4.69E-04	1.596	0.024	1.410
ENSG00000166272	*WBP1L*	5.64E-24	1.051	0.272	0.627
ENSG00000162643	*WDR63*	8.08E-04	-1.140	-0.036	-0.504
ENSG00000187260	*WDR86*	1.46E-06	1.905	0.005	0.236
ENSG00000103175	*WFDC1*	5.21E-07	-1.376	-0.439	-0.887
ENSG00000070540	*WIPI1*	4.81E-26	1.542	0.497	0.888
ENSG00000104415	*WISP1*	4.73E-20	1.562	0.052	0.488
ENSG00000114251	*WNT5A*	1.37E-10	1.138	0.057	0.352
ENSG00000075035	*WSCD2*	8.32E-19	-1.953	-0.649	-1.198
ENSG00000146457	*WTAP*	1.64E-42	2.368	0.830	1.355
ENSG00000047597	*XK*	8.54E-03	-0.855	-0.025	-1.347
ENSG00000103489	*XYLT1*	5.83E-11	1.129	0.055	0.439
ENSG00000166793	*YPEL4*	4.03E-06	-1.011	-0.102	-0.356
ENSG00000163874	*ZC3H12A*	5.33E-56	3.384	1.932	2.424
ENSG00000066185	*ZMYND12*	1.60E-05	-1.369	-0.011	-0.202
ENSG00000168661	*ZNF30*	1.15E-04	-1.093	-0.038	-0.232
ENSG00000161642	*ZNF385A*	7.29E-07	1.101	0.107	0.644
ENSG00000144331	*ZNF385B*	1.09E-04	-1.022	-0.019	-0.233
ENSG00000175322	*ZNF519*	7.13E-05	-1.299	-0.038	-0.375
ENSG00000176472	*ZNF575*	1.39E-05	-1.149	-0.033	-0.439
ENSG00000178882	*ZNF664-RFLNA*	2.75E-04	-1.472	-0.038	-0.755
ENSG00000143067	*ZNF697*	5.77E-09	1.141	0.063	0.670
ENSG00000164684	*ZNF704*	7.74E-11	-1.126	-0.187	-0.626
ENSG00000197385	*ZNF860*	2.75E-07	-1.183	-0.024	-0.839
ENSG00000132003	*ZSWIM4*	9.33E-10	1.152	0.078	0.566

Supplementary Table 5. Overview of differentially expressed genes in end-stage osteoarthritis-derived synovial fibroblasts between vehicle control and IL-17 treatment (10 ng/ml) with an adjusted p-value of <0.05. Green boxes note logarithmic fold changes (LFC) of at least +1 and red boxes note LFC of at least -1, while blank boxes show an LFC -1 and 1. Genes are sorted alphabetically on SYMBOL gene name.

**Ensemble gene name**	**SYMBOL gene name**	**Adjusted p-value**	**Log2 Fold Change (LFC)**		
			IL-17A	IL-17F	IL-17AF
ENSG00000198691	*ABCA4*	1.41E-02	1.608	0.017	0.043
ENSG00000129048	*ACKR4*	5.44E-07	-1.132	-0.024	-0.075
ENSG00000068366	*ACSL4*	9.93E-17	1.072	0.213	0.352
ENSG00000159251	*ACTC1*	1.15E-08	-1.709	-0.823	-0.968
ENSG00000138316	*ADAMTS14*	1.06E-11	-1.173	-0.374	-0.541
ENSG00000158859	*ADAMTS4*	9.61E-03	1.363	0.009	0.040
ENSG00000111452	*ADGRD1*	2.21E-11	1.993	0.041	0.900
ENSG00000150594	*ADRA2A*	6.37E-04	-1.397	-0.017	-0.053
ENSG00000111863	*ADTRP*	5.40E-04	1.027	0.047	0.505
ENSG00000042286	*AIFM2*	2.05E-16	1.192	0.445	0.637
ENSG00000154027	*AK5*	3.95E-03	-1.120	-0.032	-0.074
ENSG00000171819	*ANGPTL7*	2.52E-04	-2.096	-0.020	-0.068
ENSG00000151150	*ANK3*	1.35E-06	-1.430	-0.020	-0.055
ENSG00000154856	*APCDD1*	1.24E-03	1.100	0.023	0.496
ENSG00000171388	*APLN*	6.14E-08	1.679	0.684	1.079
ENSG00000221963	*APOL6*	9.66E-10	1.078	0.555	0.682
ENSG00000165272	*AQP3*	2.16E-10	-1.920	-0.951	-1.104
ENSG00000165269	*AQP7*	8.36E-07	-1.958	-0.030	-0.786
ENSG00000103569	*AQP9*	2.07E-10	3.261	0.018	1.766
ENSG00000106819	*ASPN*	6.59E-05	-1.163	-0.390	-0.592
ENSG00000018625	*ATP1A2*	1.75E-02	-1.055	-0.024	-0.065
ENSG00000104043	*ATP8B4*	3.24E-04	1.082	0.416	0.666
ENSG00000140379	*BCL2A1*	2.38E-09	2.812	0.033	1.549
ENSG00000069399	*BCL3*	1.61E-38	1.728	0.849	1.037
ENSG00000100739	*BDKRB1*	5.09E-05	1.169	0.021	0.066
ENSG00000023445	*BIRC3*	1.25E-08	1.167	0.009	0.048
ENSG00000153162	*BMP6*	1.28E-06	1.711	0.024	0.101
ENSG00000166920	*C15orf48*	2.19E-25	4.851	0.024	2.328
ENSG00000145861	*C1QTNF2*	2.92E-08	-1.767	-0.036	-0.322
ENSG00000163145	*C1QTNF7*	2.41E-03	-1.068	0.006	-0.004
ENSG00000139178	*C1RL*	7.97E-26	1.331	0.943	1.039
ENSG00000198535	*C2CD4A*	8.65E-12	2.015	0.037	0.742

ENSG00000205502	*C2CD4B*	2.47E-13	2.029	0.020	1.095
ENSG00000125730	*C3*	7.08E-08	1.400	0.034	0.514
ENSG00000134830	*C5AR2*	2.21E-02	1.194	0.007	0.030
ENSG00000074410	*CA12*	9.22E-12	1.017	0.324	0.450
ENSG00000204682	*CASC10*	1.13E-03	-1.142	-0.002	-0.076
ENSG00000143318	*CASQ1*	4.27E-03	-1.458	-0.014	0.003
ENSG00000198624	*CCDC69*	4.54E-05	1.282	0.031	0.676
ENSG00000181374	*CCL13*	3.19E-03	2.551	0.004	0.022
ENSG00000108691	*CCL2*	7.03E-09	2.150	0.023	0.769
ENSG00000115009	*CCL20*	3.46E-15	6.252	0.021	0.068
ENSG00000163823	*CCR1*	1.93E-03	1.096	0.016	0.097
ENSG00000126353	*CCR7*	1.97E-05	2.089	0.018	1.022
ENSG00000004468	*CD38*	2.02E-03	1.861	0.001	0.007
ENSG00000121594	*CD80*	2.00E-03	2.310	0.009	0.017
ENSG00000163814	*CDCP1*	9.80E-14	1.655	0.592	0.903
ENSG00000138395	*CDK15*	8.80E-04	-1.863	0.001	-0.034
ENSG00000221869	*CEBPD*	8.01E-19	1.888	0.792	1.063
ENSG00000105792	*CFAP69*	1.38E-18	1.498	0.494	0.605
ENSG00000243649	*CFB*	8.25E-13	1.676	0.035	0.625
ENSG00000138135	*CH25H*	3.82E-03	2.043	0.004	0.030
ENSG00000016391	*CHDH*	1.54E-05	-1.015	-0.376	-0.096
ENSG00000064886	*CHI3L2*	8.97E-24	3.314	0.770	1.760
ENSG00000159212	*CLIC6*	2.02E-06	1.648	0.029	0.866
ENSG00000134326	*CMPK2*	1.93E-02	1.671	0.871	0.039
ENSG00000154529	*CNTNAP3B*	4.06E-03	-1.012	-0.029	-0.604
ENSG00000100473	*COCH*	3.55E-04	1.317	0.026	0.028
ENSG00000204291	*COL15A1*	7.51E-06	1.019	0.382	0.583
ENSG00000081052	*COL4A4*	2.92E-03	1.067	0.019	0.037
ENSG00000047457	*CP*	3.00E-24	1.472	0.475	0.707
ENSG00000108342	*CSF3*	8.69E-18	8.823	0.023	5.969
ENSG00000135047	*CTSL*	3.03E-30	1.443	0.659	0.865
ENSG00000163131	*CTSS*	1.35E-23	1.492	0.261	0.648
ENSG00000163739	*CXCL1*	3.52E-43	7.132	4.338	4.983
ENSG00000081041	*CXCL2*	1.76E-25	4.725	2.923	3.067
ENSG00000163734	*CXCL3*	2.85E-28	4.971	2.435	2.917
ENSG00000163735	*CXCL5*	2.70E-14	3.685	0.018	1.398
ENSG00000124875	*CXCL6*	2.68E-27	6.126	3.437	4.140
ENSG00000169429	*CXCL8*	2.64E-19	3.686	0.918	1.386
ENSG00000137869	*CYP19A1*	2.38E-06	1.844	1.032	1.569
ENSG00000172817	*CYP7B1*	2.19E-03	1.703	0.012	0.045

ENSG00000164488	*DACT2*	1.80E-04	2.179	0.019	1.661
ENSG00000123977	*DAW1*	3.19E-05	2.235	0.023	0.982
ENSG00000164825	*DEFB1*	3.48E-02	1.159	0.009	0.029
ENSG00000165507	*DEPP1*	2.11E-09	1.402	0.468	0.598
ENSG00000062282	*DGAT2*	4.02E-25	2.007	1.117	1.327
ENSG00000211448	*DIO2*	7.23E-07	1.661	0.028	0.603
ENSG00000149927	*DOC2A*	6.79E-03	-1.323	-0.012	-0.051
ENSG00000102385	*DRP2*	8.72E-06	-1.610	-0.046	-0.556
ENSG00000134769	*DTNA*	1.24E-12	1.162	0.430	0.546
ENSG00000139318	*DUSP6*	5.38E-11	1.498	0.509	0.782
ENSG00000136160	*EDNRB*	8.44E-10	1.099	0.420	0.637
ENSG00000164181	*ELOVL7*	9.27E-10	1.698	0.034	0.763
ENSG00000198018	*ENTPD7*	9.37E-09	1.030	0.092	0.282
ENSG00000133106	*EPSTI1*	6.45E-03	1.281	0.926	0.818
ENSG00000124882	*EREG*	3.28E-04	1.095	0.027	0.222
ENSG00000178752	*ERFE*	2.16E-09	1.346	0.331	0.802
ENSG00000139083	*ETV6*	3.02E-11	1.014	0.474	0.633
ENSG00000185862	*EVI2B*	3.69E-10	1.934	0.585	1.265
ENSG00000110723	*EXPH5*	7.62E-13	-1.347	-0.569	-0.886
ENSG00000181104	*F2R*	1.33E-13	-1.213	-0.052	-0.400
ENSG00000121769	*FABP3*	7.39E-12	-1.154	-0.536	-0.697
ENSG00000170323	*FABP4*	1.11E-03	-1.406	-0.028	-0.058
ENSG00000150510	*FAM124A*	2.79E-08	1.299	0.039	0.634
ENSG00000159784	*FAM131B*	1.16E-02	-1.061	-0.015	-0.065
ENSG00000147724	*FAM135B*	1.68E-03	-1.137	-0.031	-0.079
ENSG00000148541	*FAM13C*	1.77E-03	-1.198	-0.021	-0.091
ENSG00000197520	*FAM177B*	1.94E-04	1.496	0.016	0.621
ENSG00000204767	*FAM196B*	4.71E-03	1.046	0.436	0.398
ENSG00000164125	*FAM198B*	4.24E-18	1.249	0.595	0.661
ENSG00000108950	*FAM20A*	8.35E-15	1.680	0.614	0.949
ENSG00000185112	*FAM43A*	1.50E-05	-1.107	-0.041	-0.370
ENSG00000165323	*FAT3*	1.34E-07	-1.146	-0.364	-0.833
ENSG00000146267	*FAXC*	1.07E-02	-1.332	-0.002	-0.015
ENSG00000162894	*FCMR*	2.36E-03	1.275	-0.005	0.020
ENSG00000118407	*FILIP1*	4.84E-02	1.127	0.005	0.020
ENSG00000204131	*FLJ44635*	1.19E-03	-1.201	-0.020	-0.110
ENSG00000125848	*FLRT3*	1.24E-09	2.141	0.026	0.746
ENSG00000138759	*FRAS1*	7.46E-05	1.587	0.019	0.986
ENSG00000131386	*GALNT15*	1.58E-19	1.225	0.569	0.720
ENSG00000162645	*GBP2*	1.19E-05	1.262	0.025	0.618

ENSG00000131979	*GCH1*	4.03E-04	1.401	0.011	0.044
ENSG00000131459	*GFPT2*	3.48E-12	1.017	0.163	0.436
ENSG00000168546	*GFRA2*	3.87E-02	1.130	0.017	0.062
ENSG00000165474	*GJB2*	4.02E-06	1.112	0.017	0.169
ENSG00000107249	*GLIS3*	4.46E-16	1.251	0.353	0.499
ENSG00000173221	*GLRX*	2.16E-13	1.168	0.399	0.589
ENSG00000127920	*GNG11*	5.58E-23	1.276	0.582	0.763
ENSG00000167588	*GPD1*	3.10E-07	-1.705	-0.031	-0.121
ENSG00000164850	*GPER1*	6.99E-09	-1.022	-0.123	-0.132
ENSG00000158292	*GPR153*	4.90E-17	-1.122	-0.413	-0.514
ENSG00000105509	*HAS1*	2.72E-06	1.649	0.016	0.081
ENSG00000188290	*HES4*	2.65E-04	1.072	0.033	1.028
ENSG00000135245	*HILPDA*	4.57E-08	1.764	0.014	0.059
ENSG00000127124	*HIVEP3*	1.30E-07	1.548	0.726	0.538
ENSG00000108924	*HLF*	2.21E-07	-1.564	-0.833	-1.054
ENSG00000164120	*HPGD*	1.14E-05	-3.144	-0.019	-0.049
ENSG00000117594	*HSD11B1*	9.16E-15	1.219	0.466	0.628
ENSG00000169271	*HSPB3*	1.77E-03	-1.657	-0.018	-0.057
ENSG00000173641	*HSPB7*	2.12E-21	-1.279	-0.470	-0.586
ENSG00000102468	*HTR2A*	2.72E-04	1.727	0.017	0.061
ENSG00000169495	*HTRA4*	1.14E-02	1.113	0.013	0.061
ENSG00000090339	*ICAM1*	3.33E-28	1.765	0.528	0.782
ENSG00000160223	*ICOSLG*	5.86E-07	1.466	0.669	0.667
ENSG00000131203	*IDO1*	3.66E-03	2.037	0.014	0.039
ENSG00000068079	*IFI35*	3.00E-04	1.172	0.427	0.544
ENSG00000103742	*IGDCC4*	1.76E-07	-1.160	-0.255	-0.449
ENSG00000146678	*IGFBP1*	7.61E-10	1.294	0.405	0.741
ENSG00000134470	*IL15RA*	2.63E-10	1.622	0.674	0.573
ENSG00000115604	*IL18R1*	9.66E-10	1.346	0.616	0.799
ENSG00000115602	*IL1RL1*	3.15E-08	1.087	0.039	0.597
ENSG00000136689	*IL1RN*	1.69E-03	1.066	0.011	0.016
ENSG00000142677	*IL22RA1*	2.27E-02	-1.101	-0.017	-0.023
ENSG00000008517	*IL32*	3.73E-13	1.719	0.582	1.167
ENSG00000137033	*IL33*	6.84E-20	1.898	0.719	1.047
ENSG00000136696	*IL36B*	1.92E-08	1.541	0.053	1.105
ENSG00000136695	*IL36RN*	1.66E-04	1.555	0.015	0.067
ENSG00000104951	*IL4I1*	2.17E-04	1.627	0.014	1.354
ENSG00000136244	*IL6*	6.10E-23	5.532	2.586	3.071
ENSG00000168685	*IL7R*	4.60E-04	1.031	0.049	0.440
ENSG00000125347	*IRF1*	2.57E-22	1.183	0.537	0.655

ENSG00000137265	*IRF4*	2.31E-03	1.058	0.018	0.048
ENSG00000128604	*IRF5*	5.34E-03	1.522	0.019	0.042
ENSG00000091409	*ITGA6*	1.52E-12	-1.016	-0.498	-0.758
ENSG00000105855	*ITGB8*	1.79E-10	1.019	0.399	0.476
ENSG00000105639	*JAK3*	3.98E-07	1.728	0.758	0.817
ENSG00000158445	*KCNB1*	1.02E-04	-1.076	-0.020	-0.023
ENSG00000143473	*KCNH1*	8.53E-04	1.229	0.025	0.635
ENSG00000184156	*KCNQ3*	2.56E-06	1.361	0.589	0.839
ENSG00000157404	*KIT*	1.25E-05	-1.175	-0.031	-0.502
ENSG00000239474	*KLHL41*	8.44E-18	-1.099	-0.474	-0.639
ENSG00000170454	*KRT75*	9.33E-06	1.836	0.025	0.846
ENSG00000115919	*KYNU*	3.00E-17	1.320	0.632	0.813
ENSG00000179630	*LACC1*	6.12E-09	1.035	0.342	0.523
ENSG00000129988	*LBP*	5.74E-10	4.020	0.014	1.532
ENSG00000169744	*LDB2*	2.01E-22	-1.345	-0.330	-0.280
ENSG00000174697	*LEP*	5.90E-08	3.769	0.013	1.784
ENSG00000128342	*LIF*	3.24E-19	1.799	0.512	0.661
ENSG00000236882	*LINC01554*	7.50E-03	2.247	0.011	0.036
ENSG00000178734	*LMO7DN*	1.99E-04	-1.012	-0.789	-0.667
ENSG00000163431	*LMOD1*	4.54E-26	-2.020	-0.658	-0.847
ENSG00000111684	*LPCAT3*	4.25E-29	1.076	0.548	0.745
ENSG00000165379	*LRFN5*	5.92E-13	-1.251	-0.066	-0.588
ENSG00000173114	*LRRN3*	1.60E-12	-1.017	-0.627	-0.700
ENSG00000162951	*LRRTM1*	3.74E-02	1.511	0.012	0.035
ENSG00000172901	*LVRN*	2.28E-05	1.187	0.023	0.132
ENSG00000165072	*MAMDC2*	1.92E-05	-1.019	-0.032	-0.330
ENSG00000107968	*MAP3K8*	2.92E-45	1.692	1.346	1.484
ENSG00000081189	*MEF2C*	1.32E-09	-1.091	-0.308	-0.611
ENSG00000005102	*MEOX1*	3.34E-03	1.052	-0.004	0.005
ENSG00000106511	*MEOX2*	2.90E-07	-1.495	-0.031	-0.180
ENSG00000106484	*MEST*	5.39E-15	-1.346	-0.503	-0.673
ENSG00000185432	*METTL7A*	2.94E-13	-1.075	-0.401	-0.611
ENSG00000168389	*MFSD2A*	2.74E-04	1.450	0.015	0.069
ENSG00000111341	*MGP*	3.90E-13	-1.158	-0.466	-0.652
ENSG00000108960	*MMD*	2.26E-21	1.029	0.409	0.588
ENSG00000196611	*MMP1*	6.39E-18	3.020	0.876	1.393
ENSG00000166670	*MMP10*	4.41E-06	1.390	0.020	0.753
ENSG00000149968	*MMP3*	3.27E-14	2.020	0.051	0.916
ENSG00000082126	*MPP4*	2.46E-02	-1.057	-0.022	-0.068
ENSG00000072952	*MRVI1*	6.13E-14	-1.613	-0.045	-0.159

ENSG00000178860	*MSC*	1.72E-14	1.255	0.255	0.436
ENSG00000138379	*MSTN*	1.45E-09	-2.534	-0.045	-0.687
ENSG00000120149	*MSX2*	2.74E-09	1.079	0.090	0.580
ENSG00000169715	*MT1E*	1.37E-18	1.151	0.438	0.631
ENSG00000198417	*MT1F*	1.73E-02	1.132	0.007	0.031
ENSG00000125144	*MT1G*	3.17E-06	2.363	0.018	0.047
ENSG00000187193	*MT1X*	9.02E-21	1.646	0.534	0.946
ENSG00000125148	*MT2A*	9.47E-13	1.399	0.390	0.705
ENSG00000183486	*MX2*	6.01E-03	2.070	1.135	0.034
ENSG00000136449	*MYCBPAP*	4.20E-02	-1.452	-0.004	-0.009
ENSG00000034971	*MYOC*	1.07E-20	-1.110	-0.592	-0.780
ENSG00000101605	*MYOM1*	3.38E-05	-1.389	-0.036	-1.203
ENSG00000105835	*NAMPT*	2.76E-13	2.208	0.039	0.758
ENSG00000176771	*NCKAP5*	1.34E-02	-1.281	-0.012	-0.030
ENSG00000124479	*NDP*	1.92E-15	2.278	0.850	1.032
ENSG00000167604	*NFKBID*	3.08E-08	1.137	0.793	1.091
ENSG00000144802	*NFKBIZ*	2.09E-163	3.681	2.950	3.025
ENSG00000204131	*NHSL2*	1.19E-03	-1.201	-0.020	-0.110
ENSG00000166741	*NNMT*	1.46E-29	2.315	1.196	1.540
ENSG00000183691	*NOG*	2.85E-05	1.411	0.037	0.924
ENSG00000171246	*NPTX1*	1.16E-11	-1.203	-0.437	-0.680
ENSG00000182575	*NXPH3*	2.54E-04	-1.157	-0.006	-0.018
ENSG00000177989	*ODF3B*	3.64E-08	1.190	0.042	0.687
ENSG00000125510	*OPRL1*	3.71E-02	-1.259	-0.007	-0.041
ENSG00000145623	*OSMR*	2.03E-19	1.335	0.513	0.710
ENSG00000187950	*OVCH1*	9.50E-03	1.398	0.018	0.035
ENSG00000243444	*PALM2*	7.00E-07	1.195	0.059	0.614
ENSG00000138496	*PARP9*	5.02E-05	1.118	0.752	0.673
ENSG00000175426	*PCSK1*	5.06E-03	1.197	0.014	0.047
ENSG00000115252	*PDE1A*	1.13E-16	-1.013	-0.313	-0.530
ENSG00000113448	*PDE4D*	1.97E-07	2.047	0.024	0.907
ENSG00000138735	*PDE5A*	1.28E-26	-1.500	-0.452	-0.633
ENSG00000162493	*PDPN*	1.10E-32	1.809	0.745	1.046
ENSG00000109272	*PF4V1*	2.61E-02	1.309	0.001	0.031
ENSG00000114268	*PFKFB4*	1.61E-04	1.331	0.014	0.057
ENSG00000119630	*PGF*	3.04E-15	1.985	0.034	1.013
ENSG00000153823	*PID1*	3.84E-08	1.598	0.034	0.528
ENSG00000122861	*PLAU*	2.46E-04	1.060	0.045	0.748
ENSG00000115896	*PLCL1*	1.06E-13	-1.576	-0.846	-0.974
ENSG00000075651	*PLD1*	3.55E-07	1.400	0.025	0.406

ENSG00000147872	*PLIN2*	3.26E-10	1.020	0.064	0.356
ENSG00000188313	*PLSCR1*	4.31E-08	1.150	0.754	0.843
ENSG00000221866	*PLXNA4*	1.04E-03	1.046	0.013	0.099
ENSG00000141682	*PMAIP1*	8.72E-09	1.447	0.065	0.728
ENSG00000028277	*POU2F2*	6.86E-36	2.188	1.344	1.434
ENSG00000158528	*PPP1R9A*	1.64E-03	-1.531	-0.017	-0.045
ENSG00000156475	*PPP2R2B*	4.73E-05	-1.987	-0.028	-0.973
ENSG00000061455	*PRDM6*	1.51E-03	-1.144	-0.022	-0.049
ENSG00000186652	*PRG2*	2.76E-06	1.052	0.052	0.519
ENSG00000146250	*PRSS35*	8.58E-12	-1.544	-0.732	-0.766
ENSG00000140368	*PSTPIP1*	2.99E-02	0.150	0.014	1.586
ENSG00000117425	*PTCH2*	2.14E-03	-1.028	-0.022	-0.119
ENSG00000148344	*PTGES*	2.40E-13	1.121	0.075	0.366
ENSG00000122420	*PTGFR*	6.82E-26	1.698	0.727	0.921
ENSG00000073756	*PTGS2*	4.12E-09	1.958	0.036	0.659
ENSG00000163661	*PTX3*	7.13E-07	1.178	0.563	0.854
ENSG00000041353	*RAB27B*	1.26E-03	1.077	0.020	0.088
ENSG00000108551	*RASD1*	3.87E-06	1.895	0.018	0.645
ENSG00000198774	*RASSF9*	8.29E-09	-1.655	-0.727	-1.054
ENSG00000088320	*REM1*	2.15E-06	-1.622	0.000	-0.078
ENSG00000117152	*RGS4*	1.50E-06	-2.045	-0.678	-1.080
ENSG00000108370	*RGS9*	2.26E-06	-1.121	-0.389	-0.535
ENSG00000143878	*RHOB*	1.75E-07	1.491	0.025	0.503
ENSG00000072422	*RHOBTB1*	5.90E-10	-1.219	-0.297	-0.273
ENSG00000126785	*RHOJ*	2.09E-06	-1.163	-0.039	-0.076
ENSG00000079841	*RIMS1*	4.57E-04	-1.113	-0.032	-0.486
ENSG00000042062	*RIPOR3*	2.99E-14	1.877	0.466	0.738
ENSG00000169071	*ROR2*	2.80E-05	1.070	0.020	0.077
ENSG00000134321	*RSAD2*	1.48E-04	2.810	1.313	0.043
ENSG00000186907	*RTN4RL2*	1.03E-22	1.886	0.670	0.897
ENSG00000102445	*RUBCNL*	7.49E-23	1.805	0.861	1.156
ENSG00000231274	*SBK3*	3.88E-05	1.810	0.020	1.017
ENSG00000064932	*SBNO2*	2.54E-24	1.187	0.443	0.642
ENSG00000170381	*SEMA3E*	9.66E-15	-1.005	-0.325	-0.501
ENSG00000197632	*SERPINB2*	1.26E-26	2.110	0.628	1.076
ENSG00000102683	*SGCG*	3.28E-04	-1.620	-0.018	-0.545
ENSG00000164690	*SHH*	1.80E-06	1.846	0.028	1.085
ENSG00000142178	*SIK1*	1.11E-05	1.118	0.020	0.246
ENSG00000141469	*SLC14A1*	9.79E-05	-1.217	-0.021	-0.072
ENSG00000110446	*SLC15A3*	1.02E-03	1.072	0.026	0.134

ENSG00000118596	*SLC16A7*	1.80E-15	1.335	0.367	0.267
ENSG00000135917	*SLC19A3*	4.07E-17	2.331	0.577	0.831
ENSG00000137266	*SLC22A23*	1.69E-14	1.005	0.350	0.472
ENSG00000185052	*SLC24A3*	3.17E-08	1.601	0.043	0.709
ENSG00000140284	*SLC27A2*	3.29E-02	1.586	0.007	0.039
ENSG00000142583	*SLC2A5*	7.20E-24	1.277	0.506	0.798
ENSG00000169507	*SLC38A11*	2.79E-02	-1.491	-0.006	-0.028
ENSG00000104635	*SLC39A14*	9.59E-05	1.293	0.021	0.076
ENSG00000138821	*SLC39A8*	2.09E-06	2.061	0.014	0.058
ENSG00000138449	*SLC40A1*	4.85E-15	-1.246	-0.520	-0.797
ENSG00000167703	*SLC43A2*	4.18E-14	1.934	0.659	0.931
ENSG00000134802	*SLC43A3*	1.73E-17	1.011	0.307	0.486
ENSG00000003989	*SLC7A2*	3.90E-18	2.875	1.107	1.566
ENSG00000155465	*SLC7A7*	6.15E-06	1.080	0.602	0.574
ENSG00000184557	*SOCS3*	1.48E-19	1.343	0.322	0.631
ENSG00000112096	*SOD2*	6.72E-07	1.756	0.030	0.595
ENSG00000112096	*SOD2-OT1*	6.72E-07	1.756	0.030	0.595
ENSG00000137767	*SQOR*	3.26E-19	1.068	0.536	0.701
ENSG00000122862	*SRGN*	3.45E-17	1.112	0.055	0.352
ENSG00000115415	*STAT1*	3.46E-12	1.004	0.628	0.739
ENSG00000138378	*STAT4*	3.57E-13	1.978	0.967	1.474
ENSG00000159167	*STC1*	1.14E-22	2.934	1.167	1.536
ENSG00000164647	*STEAP1*	4.95E-24	1.721	0.754	0.971
ENSG00000157214	*STEAP2*	1.74E-09	1.548	0.591	0.731
ENSG00000127954	*STEAP4*	1.62E-04	1.337	0.583	0.965
ENSG00000140022	*STON2*	1.76E-04	1.055	0.396	0.331
ENSG00000128578	*STRIP2*	5.19E-16	1.702	0.558	0.844
ENSG00000170921	*TANC2*	2.21E-28	-1.005	-0.482	-0.673
ENSG00000170379	*TCAF2*	2.47E-03	1.055	0.005	0.021
ENSG00000176907	*TCIM*	7.51E-03	1.850	0.008	0.022
ENSG00000197905	*TEAD4*	1.08E-08	1.127	0.069	0.586
ENSG00000105825	*TFPI2*	2.05E-19	2.438	0.907	1.345
ENSG00000038295	*TLL1*	6.70E-07	-1.060	-0.405	-0.485
ENSG00000137462	*TLR2*	8.72E-09	2.258	0.010	0.050
ENSG00000183160	*TMEM119*	2.21E-28	-1.188	-0.414	-0.454
ENSG00000139364	*TMEM132B*	3.37E-05	1.255	0.023	0.076
ENSG00000164124	*TMEM144*	3.53E-03	1.019	0.025	0.051
ENSG00000249992	*TMEM158*	2.35E-08	1.493	0.446	0.865
ENSG00000196932	*TMEM26*	5.87E-04	-1.396	-0.022	-0.079
ENSG00000182107	*TMEM30B*	5.16E-19	-1.018	-0.332	-0.558

ENSG00000133687	*TMTC1*	2.39E-15	1.015	0.325	0.454
ENSG00000185215	*TNFAIP2*	8.69E-18	1.371	0.457	0.602
ENSG00000123610	*TNFAIP6*	4.04E-12	3.429	0.822	1.478
ENSG00000127863	*TNFRSF19*	1.64E-09	-1.236	-0.049	-0.198
ENSG00000028137	*TNFRSF1B*	1.84E-14	1.273	0.460	0.680
ENSG00000125735	*TNFSF14*	3.42E-04	2.187	0.008	0.030
ENSG00000111907	*TPD52L1*	4.27E-17	-1.066	-0.367	-0.469
ENSG00000124731	*TREM1*	3.10E-06	2.256	0.013	0.053
ENSG00000173334	*TRIB1*	2.06E-08	1.277	0.036	0.554
ENSG00000158022	*TRIM63*	1.98E-02	-1.277	-0.020	-0.047
ENSG00000104321	*TRPA1*	1.66E-03	1.046	0.020	0.083
ENSG00000182463	*TSHZ2*	5.00E-10	1.054	0.329	0.499
ENSG00000025708	*TYMP*	7.04E-39	2.023	1.057	1.337
ENSG00000162692	*VCAM1*	8.74E-07	1.198	0.035	0.191
ENSG00000170162	*VGLL2*	6.70E-03	1.047	0.013	0.084
ENSG00000119703	*ZC2HC1C*	6.46E-03	1.671	0.014	0.032
ENSG00000163874	*ZC3H12A*	2.17E-47	3.152	2.226	2.377
ENSG00000144331	*ZNF385B*	1.52E-10	-1.205	-0.315	-0.284

Supplementary Table 6. Results from GSEA Hallmark pathway analysis. (A) Overview of hallmark pathways significantly (padj<0.05) changed by IL-17 treatment, with the normalised enrichment score (NES) in **black** for an increase in NES and **red** for decreases in NES.

Pathway	Chondrocytes			Synovial fibroblasts		
	IL-17A	IL-17AF	IL-17F	IL-17A	IL-17AF	IL-17F
Allograft Rejection	2.43	2.10	1.83	2.35	1.86	1.98
Angiogenesis	1.98	2.09	2.00	1.75		1.80
Apical Junction			-1.55			
Apoptosis	2.04	1.80				
Bile Acid Metabolism				1.78		
Coagulation	2.03	2.05	1.74	1.87	1.70	
Complement	2.46	2.30	1.98	2.24	2.14	2.06
Epithelial Mesenchymal Transition	2.31	2.19	1.79	1.77	1.78	1.82
Estrogen Response Early	1.71	1.75				
Glycolysis	2.28	2.06	1.75	1.66		
Heme Metabolism		1.55				
Hypoxia	2.71	2.59	1.92	2.05	1.63	
Il2 Stat5 Signaling	2.36	1.90	1.76	1.69		1.64
Il6 Jak Stat3 Signaling	2.59	2.54	2.10	2.42	2.20	2.09
Inflammatory Response	3.20	2.88	2.28	2.88	2.37	2.26
Interferon Alpha Response	2.28	1.87		2.04	1.99	1.97
Interferon Gamma Response	2.85	2.61	2.24	2.73	2.37	2.25
Kras Signaling Dn			-1.65			
Kras Signaling Up	2.34	1.99	1.74	2.28	1.80	
Mitotic Spindle					-1.64	
Mtorc1 Signaling	2.09	2.03	1.53	1.78		
Myc Targets V1	1.56	2.02	1.81	1.70		
Myc Targets V2				1.75		
Myogenesis			-1.61	-1.97	-1.82	-1.87
Oxidative Phosphorylation		1.59				
Reactive Oxygen Species Pathway		1.70	1.77			
Tnfa Signaling Via Nfkb	3.43	3.27	2.60	2.95	2.46	2.35
Unfolded Protein Response	1.98	1.93				
Uv Response Up	2.41	2.38	1.92	1.80	-1.59	
Xenobiotic Metabolism	2.07	2.09	1.70			
